# Unfolding of an RNA G-quadruplex motif in the negative strand genome of porcine reproductive and respiratory syndrome virus by host and viral helicases to promote viral replication

**DOI:** 10.1093/nar/gkad759

**Published:** 2023-09-22

**Authors:** Puxian Fang, Congbao Xie, Ting Pan, Ting Cheng, Wei Chen, Sijin Xia, Tong Ding, Junkang Fang, Yanrong Zhou, Liurong Fang, Dengguo Wei, Shaobo Xiao

**Affiliations:** National Key Laboratory of Agricultural Microbiology, College of Veterinary Medicine, Huazhong Agricultural University, Wuhan 430070, China; The Key Laboratory of Preventive Veterinary Medicine in Hubei Province, Cooperative Innovation Center for Sustainable Pig Production, Wuhan 430070, China; National Key Laboratory of Agricultural Microbiology, College of Veterinary Medicine, Huazhong Agricultural University, Wuhan 430070, China; Hubei Hongshan Laboratory, and Interdisciplinary Sciences Institute, Huazhong Agricultural University, Wuhan 430070, China; Shenzhen Institute of Nutrition and Health, Huazhong Agricultural University, Shenzhen 518000, China; Shenzhen Branch, Guangdong Laboratory for Lingnan Modern Agriculture, Genome Analysis Laboratory of the Ministry of Agriculture, Agricultural Genomics Institute at Shenzhen, Chinese Academy of Agricultural Sciences, Shenzhen 518000, China; National Key Laboratory of Agricultural Microbiology, College of Veterinary Medicine, Huazhong Agricultural University, Wuhan 430070, China; The Key Laboratory of Preventive Veterinary Medicine in Hubei Province, Cooperative Innovation Center for Sustainable Pig Production, Wuhan 430070, China; National Key Laboratory of Agricultural Microbiology, College of Veterinary Medicine, Huazhong Agricultural University, Wuhan 430070, China; The Key Laboratory of Preventive Veterinary Medicine in Hubei Province, Cooperative Innovation Center for Sustainable Pig Production, Wuhan 430070, China; National Key Laboratory of Agricultural Microbiology, College of Veterinary Medicine, Huazhong Agricultural University, Wuhan 430070, China; The Key Laboratory of Preventive Veterinary Medicine in Hubei Province, Cooperative Innovation Center for Sustainable Pig Production, Wuhan 430070, China; National Key Laboratory of Agricultural Microbiology, College of Veterinary Medicine, Huazhong Agricultural University, Wuhan 430070, China; The Key Laboratory of Preventive Veterinary Medicine in Hubei Province, Cooperative Innovation Center for Sustainable Pig Production, Wuhan 430070, China; National Key Laboratory of Agricultural Microbiology, College of Veterinary Medicine, Huazhong Agricultural University, Wuhan 430070, China; The Key Laboratory of Preventive Veterinary Medicine in Hubei Province, Cooperative Innovation Center for Sustainable Pig Production, Wuhan 430070, China; National Key Laboratory of Agricultural Microbiology, College of Veterinary Medicine, Huazhong Agricultural University, Wuhan 430070, China; Hubei Hongshan Laboratory, and Interdisciplinary Sciences Institute, Huazhong Agricultural University, Wuhan 430070, China; Shenzhen Institute of Nutrition and Health, Huazhong Agricultural University, Shenzhen 518000, China; Shenzhen Branch, Guangdong Laboratory for Lingnan Modern Agriculture, Genome Analysis Laboratory of the Ministry of Agriculture, Agricultural Genomics Institute at Shenzhen, Chinese Academy of Agricultural Sciences, Shenzhen 518000, China; National Key Laboratory of Agricultural Microbiology, College of Veterinary Medicine, Huazhong Agricultural University, Wuhan 430070, China; The Key Laboratory of Preventive Veterinary Medicine in Hubei Province, Cooperative Innovation Center for Sustainable Pig Production, Wuhan 430070, China; National Key Laboratory of Agricultural Microbiology, College of Veterinary Medicine, Huazhong Agricultural University, Wuhan 430070, China; The Key Laboratory of Preventive Veterinary Medicine in Hubei Province, Cooperative Innovation Center for Sustainable Pig Production, Wuhan 430070, China; National Key Laboratory of Agricultural Microbiology, College of Veterinary Medicine, Huazhong Agricultural University, Wuhan 430070, China; Hubei Hongshan Laboratory, and Interdisciplinary Sciences Institute, Huazhong Agricultural University, Wuhan 430070, China; Shenzhen Institute of Nutrition and Health, Huazhong Agricultural University, Shenzhen 518000, China; Shenzhen Branch, Guangdong Laboratory for Lingnan Modern Agriculture, Genome Analysis Laboratory of the Ministry of Agriculture, Agricultural Genomics Institute at Shenzhen, Chinese Academy of Agricultural Sciences, Shenzhen 518000, China; National Key Laboratory of Agricultural Microbiology, College of Veterinary Medicine, Huazhong Agricultural University, Wuhan 430070, China; The Key Laboratory of Preventive Veterinary Medicine in Hubei Province, Cooperative Innovation Center for Sustainable Pig Production, Wuhan 430070, China

## Abstract

G-quadruplex (G4) is a unique secondary structure formed by guanine-rich nucleic acid sequences. Growing studies reported that the genomes of some viruses harbor G4 structures associated with viral replication, opening up a new field to dissect viral infection. Porcine reproductive and respiratory syndrome virus (PRRSV), a representative member of *Arteriviridae*, is an economically significant pathogen that has devastated the swine industry worldwide for over 30 years. In this study, we identified a highly conserved G-rich sequence with parallel-type G4 structure (named PRRSV-G4) in the negative strand genome RNA of PRRSV. Pyridostatin (PDS), a well-known G4-binding ligand, stabilized the PRRSV-G4 structure and inhibited viral replication. By screening the proteins interacting with PRRSV-G4 in PRRSV-infected cells and single-molecule magnetic tweezers analysis, we found that two helicases, host DDX18 and viral nsp10, interact with and efficiently unwound the PRRSV-G4 structure, thereby facilitating viral replication. Using a PRRSV reverse genetics system, we confirmed that recombinant PRRSV with a G4-disruptive mutation exhibited resistance to PDS treatment, thereby displaying higher replication than wild-type PRRSV. Collectively, these results demonstrate that the PRRSV-G4 structure plays a crucial regulatory role in viral replication, and targeting this structure represents a promising strategy for antiviral therapies.

## Graphical Abstract

**Graphical Abstract:**
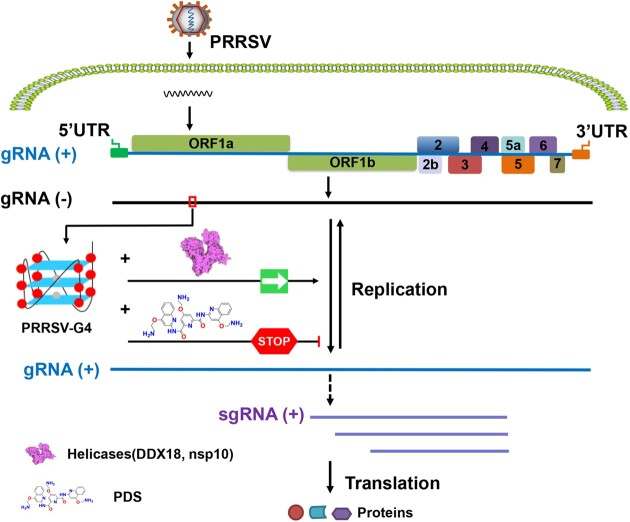

## INTRODUCTION

The G-quadruplex (G4) is a G-rich nucleotide sequence that arises from the stacking of a G·G·G·G tetrad (G-tetrads) in DNA and RNA (1). Since the first characterization of G4, numerous studies have demonstrated their roles in biological processes, such as DNA replication, gene expression and regulation, telomere maintenance, and genome stability (2–5). G4s also particulate in cellular processes through interactions with proteins, especially helicases (6). While considerable studies on the interaction of DNA G4s with proteins have been reported, only few reports have been published on proteins that bind RNA G4s (6–8).

Multiple studies on G4 structures in DNA viruses have shed light on the role of G4s in modulating viral translation and replication (9–13). Genome-wide screening in the human cytomegalovirus (HCMV), human papillomavirus (HPV), and adenovirus genome demonstrated that conserved G-rich sequences present in the virus genomes form G4 structures and modulate viral replication (9,14,15). The terminal repeat region of Kaposi's sarcoma-associated herpesvirus (KSHV) genome contains a GC-rich DNA element confirmed to form G4 structures, which are associated with possible induction of the stalling of replication forks (11). The presence of G4 structures in herpes simplex virus 1(HSV-1) genome and elucidation of their function on the viral life cycle were extensively reported (16–18). G4s in nuclear antigen 1 (EBNA1) of Epstein–Barr virus (EBV) has been confirmed to regulate EBNA1 protein synthesis and the presentation of EBNA1-specific CD8^+^T cell epitopes, involving in persistent infections (10,19). Several studies showed that G4 structures present in the pre-core promoter region and envelope gene promoter of hepatitis B virus (HBV) regulate transcription and virion secretion (20,21). In contrast to the number of studies on DNA viruses, there are very few reports on G4 structures in RNA viruses, such as human immunodeficiency virus-1(HIV-1) (22,23), hepatitis C virus (HCV), Ebola virus, Nipah virus (NiV), and severe acute respiratory syndrome coronavirus 2 (SARS-CoV-2) (24–27). A recent study demonstrated the presence of RNA G4s in HIV-1 genome and the G4 binders inhibits the initiation of viral reverse transcription (28). Notably, studies on G4 structures in viruses and the ligands targeting the G4 structures have emphasized the potential of G4 structures as novel anti-viral targets (29). Therefore, understanding G4-mediated regulation of viral replication may help contribute to the development of novel therapeutic interventions.

Porcine reproductive and respiratory syndrome (PRRS) is one of the most significant diseases that affect the global pig industry. Porcine reproductive and respiratory syndrome virus (PRRSV), which belongs to the family *Arteriviridae* of the order *Nidovirales*, is a non-segmented, positive-sense single-stranded RNA virus with a full-length genome approximately 15 kb in length (30,31). The replication of PRRSV is similar to that of coronavirus and involves the synthesis of the negative strand RNA intermediate, followed by the production of new genomic RNAs, a series of sub-genomic RNAs, and subsequent expression of viral structural protein (32). As a single-stranded RNA virus, PRRSV is prone to undergo genomic mutations that play a crucial role in viral evolution; this has led to great challenges in the development of broad-spectrum antiviral drugs and vaccines for PRRSV (33–36). Many commercial vaccines against PRRSV have been applied in the swine industry, but they do not provide durable defense against PRRSV (37,38). Thus, the identification of novel and effective therapeutic strategies targeting PRRSV is urgently required.

In this study, we identified a highly conserved G-rich sequence with a parallel-type G4 structure, PRRSV-G4, in the negative strand genome RNA of PRRSV. Pyridostatin (PDS), a G4 ligand, stabilized the PRRSV-G4 structure and exerted a significant anti-PRRSV effect. We found that host DDX18 and viral nsp10 helicases interact with and unwound the G4 structure, thereby facilitating viral replication. Recombinant PRRSV with a G4-disruptive mutation exhibited resistance to PDS treatment. These findings provide a basis for further research into potential anti-PRRSV drugs focused on targeting RNA G4 structures and helicases.

## MATERIALS AND METHODS

### Cells, virus, and reagents

PK-15^CD163^ cells, PK-15 cells stably expressing the PRRSV receptor CD163, and MARC-145 cells were cultured in Dulbecco's modified Eagle's medium (DMEM) (Invitrogen, USA) supplemented with 10% fetal bovine serum (FBS) at 37°C in a humidified atmosphere containing 5% CO_2_. The immortalized porcine alveolar macrophage (PAM) cell line, iPAM cells, which is highly susceptible to PRRSV, were cultured in RPMI 1640 medium (Sigma, USA) supplemented with 10% heat-inactivated FBS at 37°C in a humidified atmosphere containing 5% CO_2_. The PRRSV WUH3 strain (GenBank number: HM853673.2) is a highly pathogenic type 2 (North American) virus that was isolated in 2006 from the brains of pigs with ‘high-fever syndrome’ in China (39). Experiments involved in PRRSV infection were performed in the context of biosafety level 2 (BSL-2). iPAM or MARC-145 cells in T175 cm^2^ tissue culture flasks were infected with PRRSV(MOI = 1) for 1 h. After two washes with serum-free RPMI 1640 medium or DMEM to remove non-internalized virions, the cells were cultured until the occurrence of obvious cytopathic effects. These cells were frozen and thawed three times, and then centrifuged at 4 °C 12000 rpm for 10 min, and the supernatants were collected for virus stocks. RNA oligonucleotides were obtained from GenScript Company (NJ, USA) with HPLC purification and dissolved in nuclease-free water (ThermoFisher Scientific, USA) to the concentration of 100 μM. ssRNA Ladder marker (Code No. 3416) was purchased from TakaRa-Bio (Dalian, China). PDS (HY-15176A) and Lovastatin (HY-N0504) was purchased from MedChemExpress (MCE, NJ, USA). All stock and buffer solutions were prepared with nuclease-free water. Mouse anti-Flag, -hemagglutinin (HA) and rabbit anti-GFP antibodies were purchased from Medical and Biological Laboratories (MBL, Nagoya, Japan). Cell counting kit-8 (CCK-8) and BeyoClick™ EdU cell proliferation assay kits (TMB) were purchased from Beyotime (Shanghai, China).

### Identification of the putative G4 and conservation analysis

Genome-wide prediction of putative G4s in the PRRSV WUH3 strain was performed using online G4 prediction tools, including QGRS Mapper, (http://bioinformatics.ramapo.edu/QGRS/index.php/) (40), G4 Hunter (https://bioinformatics.cruk.cam.ac.uk/G4Hunter/README.html) and pqsfinder (https://pqsfinder.fi.muni.cz/). The G4 sequence was defined as G_≥3_N_1-7_G_≥3_N_1-7_G_≥3_N_1-7_G_≥3_, where G referred to guanine and N referred to any nucleotide including guanine. This algorithm was used to identify G4s in both the sense and anti-sense strands of the viral genome. Putative G-quadruplex sequences in PRRSV RNA analyzed by QGRS Mapper, G4 Hunter and pqsfinder analysis tools were shown in Table 1. For G4 conservation analysis, PRRSV genome sequences were retrieved from the National Center for Biotechnology Information website (NCBI, https://www.ncbi.nlm.nih.gov/), and multiple sequence alignment of the G4 was carried out with DNAMAN software. The graphical representation of the conserved G4 sequence was created using the WebLogo software program, version 2.8.2 (http://weblogo.berkeley.edu/) (41).

**Table 1.** Putative G-quadruplex sequences in PRRSV (-) RNA analyzed by QGRS Mapper, G4 Hunter and pqsfinder analysis tools. HCV G4 sequence serves a control in this study

			G-score		
Name	G-rich sequences (5′ to 3′)	Length	QGRS Mapper	G4 hunter	pqsfinder
G4-1	GGGCCGGUACGGGAGGGGGCAGGG	24	37	1.875	60
G4-2	GGGUGGGGGUGCGGGGGUUGGG	22	41	2.636	63
HCV G4	GGGUUGCGGGUGGGCGGG	18	39	1.944	69

### Circular dichroism (CD) spectroscopy

The sequences of RNA oligonucleotides used for CD spectroscopy are listed in Table 2. RNA samples were dissolved in 10 mM Tris–HCl buffer (pH 7.4) supplemented with different concentrations of K^+^ ion with or without 4 mM MgCl_2_. The samples were denatured at 95°C for 5 min and then slowly annealed to room temperature overnight. CD spectroscopy experiments were carried out at 25°C on a Jasco-J1500 spectropolarimeter (Jasco Hachioji, Tokyo, Japan) with a 1 mm optical path length quartz cuvette. CD spectra were recorded from 320 to 200 nm with a scanning speed of 100 nm/min; each spectrum represents the average of three accumulations (bandwidth = 1.0 nm, response time = 1.0 s, data pitch = 1.0 nm). All samples of the spectra were baseline-corrected for the signal contributions by buffer. For CD melting analysis, CD-melting measurements were recorded on a Jasco-J1500 spectropolarimeter equipped with a water bath accessory to control temperature (heating rate = 1°C/min). The CD ellipticity at 260 nm was measured as a function of temperature. The recorded temperature range was set from 15°C to 90°C.

**Table 2.** Synthetic sequences of G4 RNAs and their mutants

Name	Sequences (5′ to 3′)
PRRSV-G4	GGGUGGGGGUGCGGGGGUUGGG
PRRSV-G4 mut	GAGUGAAAGUGCGAAAGUUGAG
PRRSV-G4 mut1	GGGUGUAAAUGCGCGAGUUGGG
PRRSV-G4 mut2	AAGUGGGGGUGCGGGGGUUGUA
PRRSV-G4 mut3	AAGUGUAAAUGCGCGAGUUGUA
HCV G4	GGGUUGCGGGUGGGCGGG
HCV G4 mut	GAGUUGCGAAUGAGCAAG

### Native polyacrylamide gel electrophoresis (PAGE)

RNA samples were prepared at a concentration of 5 μM in a buffer (10 mM Tris–HCl [pH 7.4], 1 mM EDTA) containing 100 mM KCl, heated to 95°C for 5 min, and then gradually cooled to room temperature. Native polyacrylamide gels with a high concentration (20%) of acrylamide were prepared with 1 × Tris Borate EDTA (TBE) buffer, and approximately 5 μl of RNA (25 pmol) was loaded. Native PAGE was performed in 1 × TBE containing 100 mM KCl at 100V at 4°C for 4 h. SYBR Gold (ThermoFisher Scientific, USA) diluted 10 000-fold with TBE buffer was used to stain gels after electrophoresis, and bands were detected using the SYSTEM Gel Doc XR+ (Bio-Rad, USA). Oligomers were visualized by staining, and images were obtained.

### ^1^H nuclear magnetic resonance (NMR) spectroscopy

The one-dimensional ^1^H NMR spectra were mostly recorded at 308 K on Bruker 700-MHz spectrometers equipped with a regular probe. RNA samples were dissolved in 5 mM Potassium Cacodylate (Kcaco) (pH 6.5), 50 mM KCl, and 10% D_2_O at a final concentration of 300 μM. The NMR data were processed with Bruker TopSpin Version 3.2 and MestReNova. Formation of the G4 structure was demonstrated by the presence of an imino proton peak in the 10–11.5 ppm region of the ^1^H NMR spectrum, which is highly characteristic of the Hoogsteen hydrogen bonds of G4 (42).

### Single-molecule magnetic tweezer experiment

A flow chamber was established on a (3-aminopropyl) triethoxy silane (APTES; Sigma-Aldrich) functionalized coverslip 32 × 24 mm in size. The thiol-end of DNA was covalently attached to the amine group of APTES through a sulfo-SMCC crosslinker (ThermoFisher Scientific). The chamber was then blocked with BSB solution (1 × phosphate buffered saline buffer containing 2% BSA, 0.02% Tween-20, and 0.1 g/l NaN_3_) before introducing 2.7 μm diameter streptavidin-coated paramagnetic beads. Unfolding and refolding assays were carried out at 22°C in a buffer of 10 mM Tris–HCl (pH 8.0), 100 mM KC1, 4 mM MgCl_2_ and 1 U/μl, Ribolock RNase inhibitor (EO0382, ThermoFisher Scientific, USA). The extension change of the construct was detected from the diffraction pattern of the bead recorded using a CCD camera that acquired ∼200 frames per second. Force was controlled by changing the distance between the permanent magnets and the functionalized flow chamber. The magnetic tweezers have a spatial resolution for beads stuck on the surface of ∼2 nm, and the force calibration has a relative error of <10%. All experiments were carried out at 22°C, and the temperature was controlled by a thermostat. The unfolding and refolding of the G4 structures were reflected by the abrupt change of the height of the magnetic ball.

### Plasmids and EGFP expression assay

The PRRSV-G4 sequence was cloned into the N-terminus of the EGFP reporter gene of the pEGFP-C1 vector. The forward primer was designed to contain a pendant 5′-segment comprising a NheI cleavage site and PRRSV-G4, along with the 3′ region that perfectly matches the N terminus of EGFP in frame. The previously reported HCV G4 RNA and HCV G4 mutant (Table 2) were used as controls (25). The reverse primer C1-R was designed to include a HindIII cleavage site at the 5′ end and the 3′ region perfectly matches the C terminus of EGFP. The primers are listed in Table 2. The pEGFP-PRRSV-G4 and the mutant pEGFP-PRRSV-G4 mut were generated by the same way. All constructs were confirmed by Sanger sequencing analysis.

iPAM cells were transfected with plasmids using Lipofectamine 2000 (Invitrogen, USA), followed by treatment with 10 μM PDS for 12 h. The cells were fixed with 4% paraformaldehyde for 15 min, permeabilized with 0.5% Triton X-100 for 10 min, and treated with primary antibody, Alexa Fluor 488-conjugated second antibody, and subsequent 4ʹ,6-diamidino-2-phenylindole (DAPI) for 15 min. Cells were imaged by fluorescence microscopy. EGFP expression was quantified by Gen 5 software (BioTek, USA).

### Plasmid construction and protein expression and purification

The fully sequenced porcine DDX18 cDNA was obtained from PK-15^CD163^ cells and cloned into the pCAGGS-Flag construct with an N-terminal Flag tag or the pET-30a vector. The nsp10 gene of the PRRSV WUH3 strain was amplified and cloned into the pCAGGS-HA or pET-30a vector. The resulting recombinant plasmids were named pCAGGS-Flag-DDX18, pET-30a-DDX18, pCAGGS-HA-nsp10 or pET-30a-nsp10. Based on previous reports (43–45), we constructed the plasmid pET-30a-DDX18-K345E with a mutation of Lys345Glu encoding potential ATPase and helicase-inactive DDX18 (DDX18-K345E). The plasmid pET-30a-nsp10-E226A encoding ATPase and helicase-inactive nsp10 (nsp10-E226A) was gifted by Dr. Guiqing Peng at Huazhong Agricultural University (46). All primers are listed in Supplementary Table S1. Recombinant plasmids pET-30a-DDX18/DD18-K345E, pET-30a-nsp10/nsp10-E226A and the empty vector pET30a, pGEX-6P-1 were separately transformed into BL21 (DE3) cells; the cells were then induced under 0.8 mM IPTG at 18°C for 18 h. Recombinant wild-type or mutated DDX18 and nsp10 proteins were purified as described previously (46). Briefly, the cell supernatant of broken bacteria was loaded onto a Ni-NATA-His Trap HP column (GE Healthcare, USA), and proteins were eluted with elution buffer (20 mM Tris–HCl, 500 mM NaCl, 500 mM imidazole [pH 7.4]). The protein samples were then concentrated to approximately 2.0 ml and filtered using a Superdex 200 gel filtration column (GE Healthcare, USA) equilibrated with buffer (20 mM Tris–HCl and 200 mM NaCl [pH 7.4]). The concentration of purified proteins was determined using a NanoDrop 2000c UV-Vis spectrophotometer (ThermoFisher Scientific, USA). All proteins were stored at −80°C until use without freeze and thaw cycles.

### Generation of recombinant viruses

Combining the established PRRSV reverse genetics system (in preparation) and CRISPR/Cas9 technology (47), we constructed recombinant PRRSV with the G4-disruptive mutation. Briefly, two specific primers (sgPRRSV-1 and sgPRRSV-2) targeting the upstream and downstream sequences of the fragment of interest were designed and synthesized to generate sgRNA-1 and sgRNA-2. pCMV-WUH3 was then cleaved into the linearized pCMV vector by the *in vitro* addition of Cas9, sgRNA-1, and sgRNA-2. Fragments containing each of the PRRSV-G4 with the indicated mutation were generated through overlapping PCR using the indicated primers (Supplementary Table S1) and ligated into the purified linearized pCMV vector by homologous recombination to generate plasmids (pCMV-WUH3-G4 mut1, pCMV-WUH3-G4 mut2, pCMV-WUH3-G4 mut3). These recombinant plasmids were transfected into MARC-145 cells seeded in 6-well plates with 80% confluence using jetPRIME (Polyplus, Illkirch, France) for 4 h. The cells were washed twice and then incubated in DMEM containing 2% FBS at 37°C and 5% CO_2_. Daily observations were made. Upon the occurrence of an obvious cytopathic effect (CPE) that was featured by swelled, rounded, clustered and deciduous cells, transfected cells were harvested and frozen and thawed three times, followed by passage and identification of recombinant virus.

### Virus titrations by 50% tissue culture infectious dose (TCID_50_) assay

Briefly, monolayers of MARC-145 cells seeded in 96-well plates were washed twice with DMEM with 2% FBS. Virus-containing supernatants were serially diluted tenfold, and appropriate dilutions of virus suspension were chosen to inoculate cells, with eight replicates at each dilution. After the cells were cultured at 37 °C with 5% CO_2_ for 4–7 days, the TCID_50_ was calculated by the Reed-Muench method (48).

### Western blot analysis

Protein samples were separated by SDS-PAGE (12% poly-acrylamide) and transferred onto a polyvinylidene difluoride membrane (Millipore, USA). The membrane was blocked with 5% bovine serum albumin (BSA) in washing buffer (0.05% Tween in PBS) and then probed with the indicated primary antibodies overnight at 4°C. After removing antibodies, the membrane was washed three times and incubated with HRP-conjugated secondary antibodies (Beyotime, China). After washing three times, the bands were visualized by enhanced chemiluminescence reagents (Bio-Rad, USA).

### RNA extraction and reverse transcription quantitative real-time PCR (RT-qPCR)

RNA was extracted from PRRSV-infected cells using TRIzol reagent (Invitrogen, USA) and the concentration of extracted RNA was determined by a nano-drop spectrophotometer. For cDNA synthesis, only RNA with an A260/280 ratio between 1.9 and 2.1 was selected. Total RNA (1 μg) from each sample was reverse transcribed to cDNA using the Transcriptor First Strand cDNA Synthesis Kit (Roche, Switzerland), with the following reaction conditions: 55°C for 30 min and then 85°C for 5 min. The quantitative PCR reaction was performed following the minimum information for publication of quantitative real-time PCR experiments (MIQE) guidelines (49). The above cDNA (1μl) was used as template to perform RT-qPCR using Taq Pro Universal SYBR qPCR Master Mix (Vazyme Biotech Co.) in an ABI 7500 real-time PCR system (Applied Biosystems, USA). The reaction volume contains 1μl of cDNA, 0.2 μl of specific forward/reverse primers, 5 μl of 2 × Taq Pro Universal SYBR qPCR Master Mix and 3.6 μl of RNase-free H_2_O, followed by qPCR according to the thermal profile (95 °C for initial 30 s, followed by 40 cycles of 95°C for 10 s and 60°C for 30 s). For an absolute qPCR, gene mRNA levels were calculated using standard curves. For relative qPCR, gene mRNA expression levels were normalized to the transcription of glyceraldehyde-3-phosphate dehydrogenase (GAPDH) as reference genes. The results are expressed as fold change using the threshold cycle (2^−ΔΔCT^) formula and the efficiencies of qPCR assays were between 90% and 110%. Analysis of the results was done using QuantStudio™ Real-Time PCR software (Applied Biosystems, USA). The negative and positive strand genome RNA of PRRSV were quantified at 5′UTR (5′ untranslated region) and nucleocapsid (N) gene using primers (5′UTR-F/R, ORF7-F/R) reported previously (50,51). The qPCR primers are synthesized by Sangon Biotech Corporation (Wuhan, China) and listed in Supplementary Table S1.

### Indirect immunofluorescence assay (IFA)

Cells seeded in 24-well dishes were transfected or treated with PDS and infected with PRRSV for 1 h at 37°C. After two washes with serum-free RPMI 1640 medium or DMEM, the cells were cultured with RPMI 1640 medium or DMEM with 2% FBS in the absence or presence of corresponding inhibitors. At the indicated time post-infection, the cells were fixed with 4% paraformaldehyde for 15 min and then permeabilized with methyl alcohol for 10 min. After three washes with PBS, the cells were blocked with 5% BSA and incubated with mouse anti-PRRSV N protein antibody and Alexa Fluor 488-labeled anti-mouse secondary antibody (Santa Cruz Biotechnology, USA) for 1 h. Nuclei were stained with DAPI for 15 min. Fluorescent images were examined using an inverted fluorescence microscope (Olympus IX73).

### Streptavidin pull-down assays

PK-15^CD163^ cells seeded in 150 mm dishes were infected with PRRSV at a multiplicity of infection (MOI) of 2 or untreated for 24 h. The cells were washed with PBS and lysed with 250 μl of cytoplasmic lysis buffer (25 mM HEPES, 5 mM KCl, 0.5 mM MgCl_2_, 0.5% (v/v) NP-40, pH 7.9) containing protease inhibitor (Beyotime, China), phosphate inhibitor cocktail, and Ribolock RNase inhibitor for 5 min on ice, followed by centrifugation at 5000 rpm for 5 min at 4°C. The supernatant was transferred to a new tube on ice and the pellet was lysed with 250 μl of nuclear lysis buffer (25 mM HEPES, 10% (w/v) sucrose, 350 mM NaCl, 0.01% (v/v) NP-40, pH 7.9) including protease inhibitor, phosphate inhibitor cocktail, and Ribolock RNase inhibitor. Next, 500 nM of 5′-biotin labelled PRRSV-G4 was added to the mixture containing nuclear and cytoplasmic fractions; the sample was incubated for 30 min on a rotating wheel, followed by the addition of 40 μl of streptavidin agarose beads (ThermoFisher Scientific, USA) for another 30 min at room temperature. The beads were washed three times with cytoplasmic lysis buffer and nuclear lysis buffer. The harvested protein samples were subjected to the SDS-PAGE and silver staining analysis.

### LC–MS/MS analysis

Briefly, protein samples (50 μg) in UA buffer (8 M Urea, 150 mM Tris–HCl pH 8.0) were added with DTT and iodoacetamide to reduce and block the cysteine residues, followed by addition of trypsin for digestion at 37°C for 20 h. Each fraction was injected for Nano LC–MS/MS analysis on a Q Exactive mass spectrometer (ThermoFisher Scientific) coupled to Easy nLC (Proxeon Biosystems, ThermoFisher Scientific). The peptide mixture was added into a reverse phase trap column connected to the C18-reversed phase analytical column in buffer A (0.1% Formic acid), followed by separation with a linear gradient of buffer B (84% acetonitrile and 0.1% Formic acid). The flow rate is 300 nl/min and modulated by IntelliFlow technology. MS data was obtained by a data-dependent top20 method that dynamically selected the most abundant precursor ions from the survey scan (300–1800 m/*z*) for HCD fragmentation. Automatic gain control (AGC) target, maximum inject time, number of scan ranges and dynamic exclusion duration were set to 1e6, 50 ms, 1 and 30 s, respectively. Survey scans were obtained at a resolution of 70 000 at *m*/*z* 100. Resolution for HCD spectra and isolation width were set to 17500 at *m*/*z* 100 and 1.5 *m*/*z*, respectively. Normalized collision energy and the underfill ratio were set to 27 eV and 0.1%, respectively. MASCOT engine (Matrix Science, London, UK; version 2.2) embedded into Proteome Discoverer 1.4 was used to search MS/MS spectra. For protein identification, the following conditions were used. Peptide mass tolerance = 20 ppm, MS/MS tolerance = 0.1 Da. The LC–MS/MS was carried out by Shanghai Applied Protein Technology Co. Ltd.

### CCK-8 and EdU assays

Monolayer of iPAM cells seeded to 96-well cell culture plates were treated with different concentrations of drug inhibitors for 12 h. Then, 10 μl CCK-8 solution (Beyotime, China) were added into per well and incubated at 37°C for 2 h, followed by the detection of optical density at 450 nm in each well via a microplate spectrophotometer. Cell proliferation was assessed using the EdU cell proliferation assay kit with TMB (Beyotime), which is based on EdU as a novel alternative for 5-bromo-2′-deoxyuridine (BrdU) assay to directly measure active DNA synthesis or S-phase synthesis of a cell cycle (52,53), according to the manufacturer's protocol. The absorbance was detected at 370 nm and calculated as a ratio against control group cells.

### Statistical analysis

Statistical significance was determined by Student's *t*-test or one-way or two-way analysis of variance using GraphPad Prism 6 software. *P* values <0.05 were considered statistically significant.

## RESULTS

### Bioinformatic analysis of the PRRSV genome

**Figure 1.**
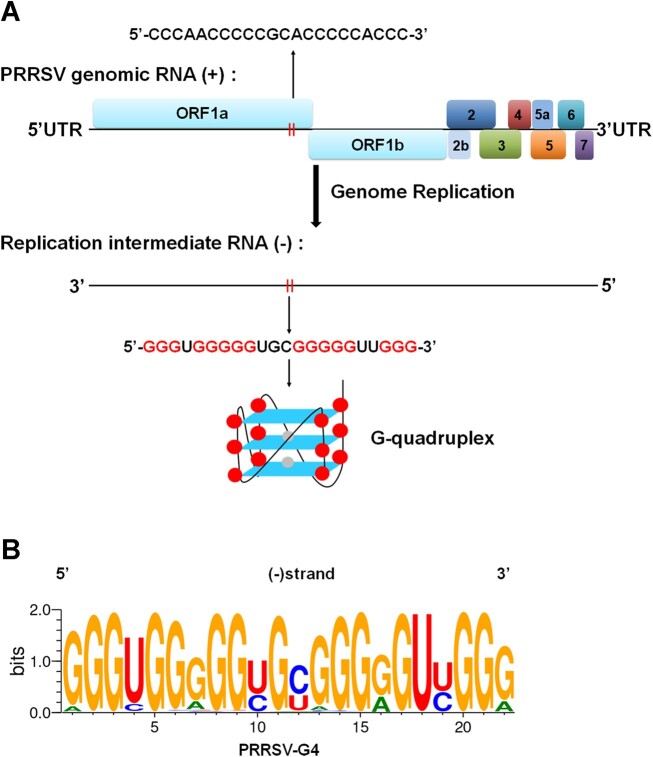
Graphical representations of the consensus G-rich sequence in the negative strand genome RNA of PRRSV. (**A**) Schematic representation of a G-rich sequence, named PRRSV-G4, in the negative strand genome RNA of PRRSV. (**B**) A total of 775 PRRSV complete genomes were retrieved from the NCBI website (https://www.ncbi.nlm.nih.gov/) and aligned. The graphical representation of PRRSV-G4 was created using WebLogo software.

Through systematic bioinformatics analysis, we identified two G-rich sequence (G4-1 and G4-2) present in the negative strand genome RNA of PRRSV that fulfilled the criteria for G4 formation (G_≥3_N_1-7_G_≥3_N_1-7_G_≥3_N_1-7_G_≥3_, where N refers to any base); G-scores of the two potential G4 sequences, as well as HCV G4 (as a control) have been shown in Table 1. Of them, G4-2 has a highest G-scores based on the QGRS Mapper and G4 hunter analysis tools, but has a second higher G-score to that of HCV G4 based on pqsfinder analysis tool. Because all three analysis tools showed that G4-2 has a higher G-score than G4-1, we focused on the characterization of G4-2 structure and function in subsequent study. G4-2 sequence was named PRRSV-G4 (Figure 1A). Multiple sequence alignment analysis using 775 complete genomic sequences of PRRSV retrieved from the NCBI database showed that the PRRSV-G4 was highly conserved among different PRRSV strains (Figure 1B). Considering the indispensable role of the negative strand genome RNA in genomic replication of PRRSV, the high conservation of PRRSV-G4 with potential G4 formation suggested it may play an important role in the viral replication of PRRSV.

### Validation of the PRRSV-G4 structure

**Figure 2.**
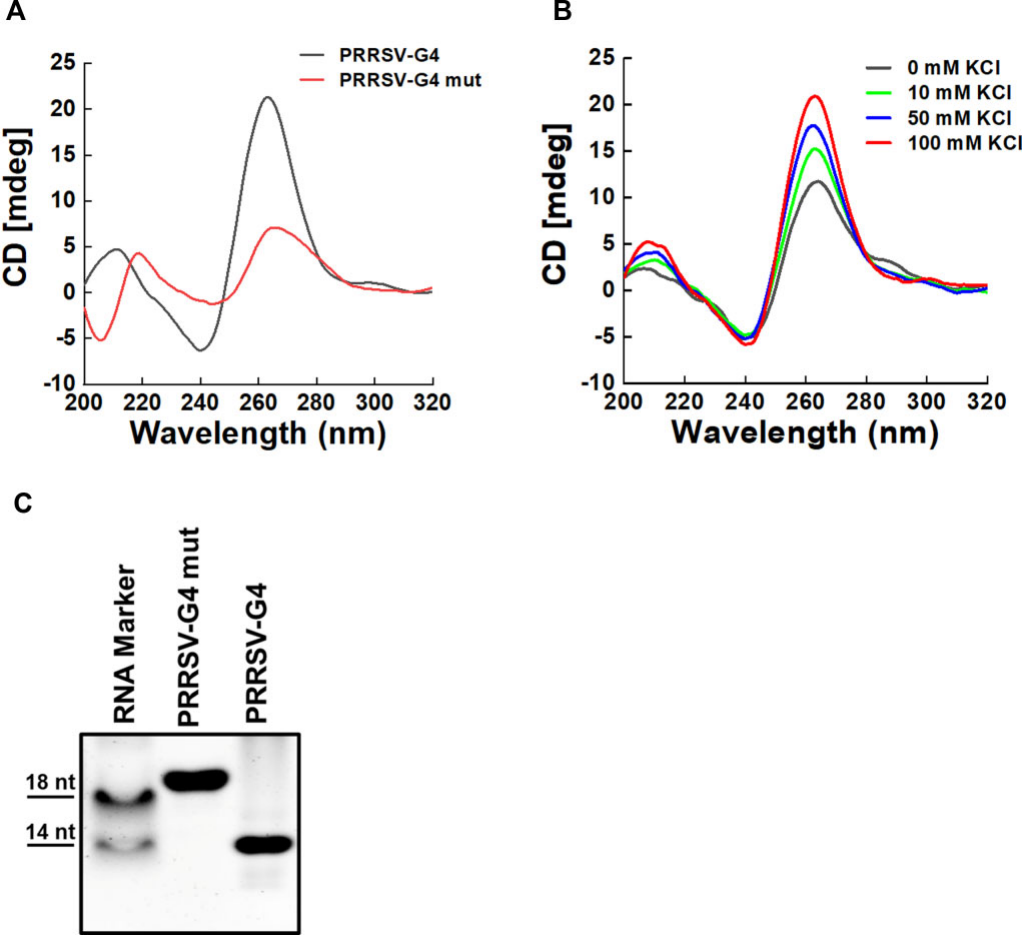
Confirmation of G4 structure formation of PRRSV-G4. (**A**) CD spectra analysis of the PRRSV-G4 wild-type and mutant (20 μM) in the presence of 100 mM K^+^ ion. (**B**) CD spectra analysis of G4 stabilization with an increasing concentration of K^+^ ion. (**C**) Native PAGE assay for characterizing the G4 structure of PRRSV-G4 wild-type and mutant. Lane 1, ssRNA markers; Lane 2, PRRSV-G4 mut; Lane 3, PRRSV-G4.

To verify formation of the PRRSV-G4 structure, we performed CD spectroscopy analysis, a dependable biophysical method to monitor G4 conformation (54). As shown in Figure 2A, the recorded CD spectra of PRRSV-G4 displayed the characteristic ∼260 nm positive peak and ∼240 nm negative peak in the presence of K^+^ ion (100 mM), indicating a parallel G4 topology. However, the PRRSV-G4 mutant (PRRSV-G4 mut) (Table 2), disrupting the G-tetrad motif necessary for G4 formation by mutating G to A, did not exhibit the CD spectral signatures associated with G4 structures. CD spectra analysis showed a gradual increase in the CD ellipticity for PRRSV-G4 with increasing concentration of K^+^ from 0 mM to 100 mM (Figure 2B). To further identify the conformation of PRRSV-G4, we performed native PAGE and ^1^H NMR analysis. Compared with the mutant counterpart, PRRSV-G4 showed a faster migration (Figure 2C). We observed a band with larger size than PRRSV-G4 structure in the uncropped native gels and speculated that it might be an intermolecular species (Supplementary data 1; Supplementary Figure S1). In addition, the NMR spectrum results showed that PRRSV-G4 exhibited imino peaks within 10–11.5 ppm region of the ^1^H NMR spectrum, while no peak was observed for PRRSV-G4 mut in this region, indicating the formation of the G4 structure (Supplementary Figure S2). Collectively, these results suggest that the PRRSV-G4 forms a stable parallel-type G4 structure in the presence of K^+^ ion.

### PDS stabilizes the PRRSV-G4 RNA structure

Recent studies have demonstrated that PDS, a representative G4 binding ligand, specifically binds and stabilizes G4 structures (10,29) (Supplementary Figure S3A). Therefore, CD melting curve analysis was performed to quantitatively evaluate the stability of PRRSV-G4 in the presence of different concentrations of PDS by monitoring the CD ellipticity at 260 nm. As shown in Supplementary Figures S3B and C, PDS treatment enhanced the stability of the PRRSV-G4 structure in a dose-dependent manner in 10 mM Tris–HCl buffer (pH 7.4) with 5 mM KCl. When the number of equivalents of PDS is increased to 3 equiv, the enhanced stability of PRRSV-G4 indicates a △*T*m of 9°C. These results showed that PDS has a stabilizing effect on the PRRSV-G4 RNA structure.

### Repression of EGFP expression through PRRSV-G4 stabilization

**Figure 3.**
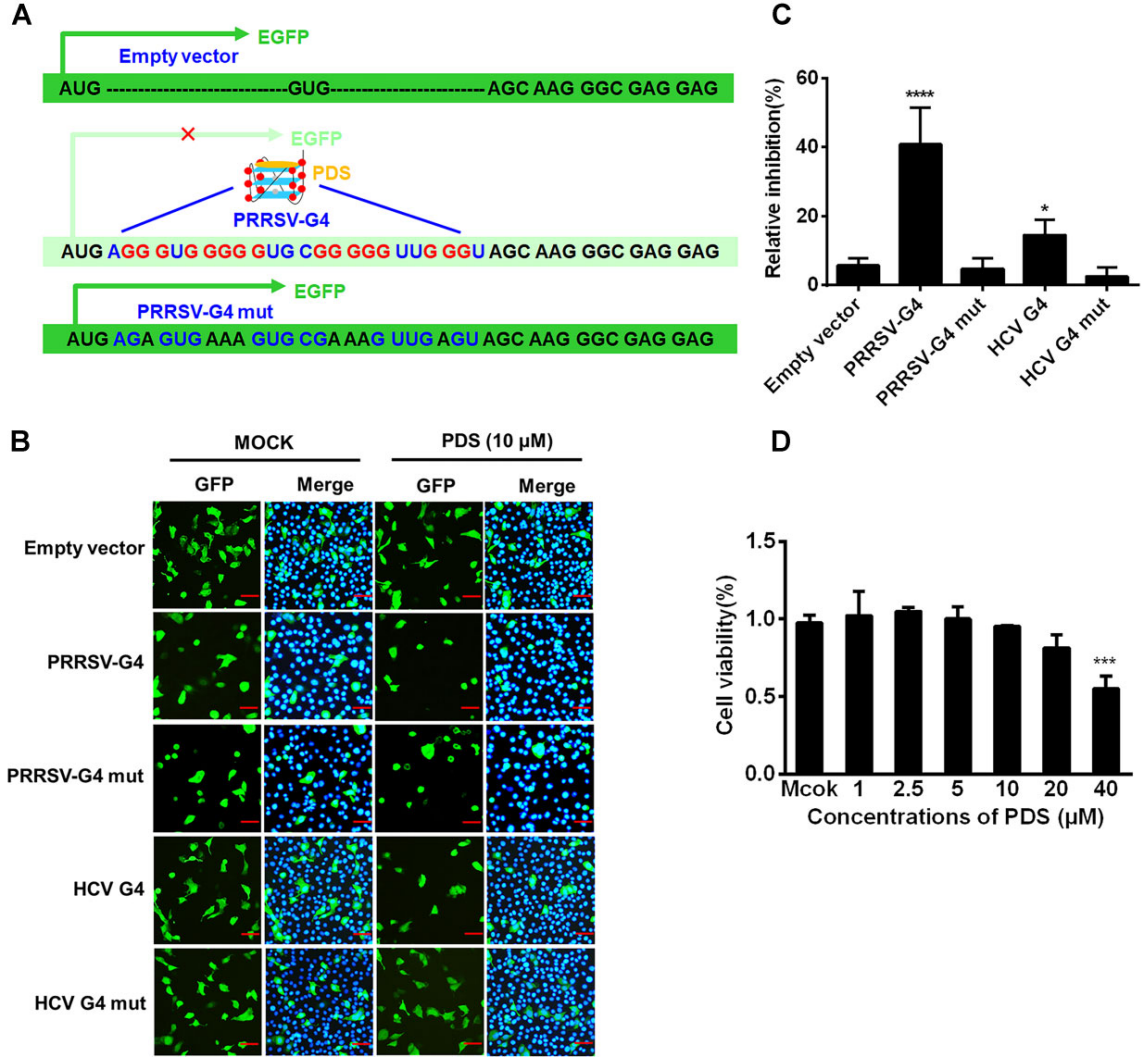
Repression of EGFP expression through PRRSV-G4 stabilization. (**A**) Schematic representation of the construction strategy for pEGFP-C1 derivatives with or without the PRRSV-G4 wild-type and mutant (PRRSV-G4 mut). The pEGFP-C1 plasmid carries the wild-type EGFP sequence; in the engineered plasmids, PRRSV-G4 or the mutant sequence was inserted upstream of the EGFP gene. (**B**) iPAM cells transfected with pEGFP-PRRSV-G4, pEGFP-HCV-G4 or mutant constructs were treated with PDS for 12 h; cells were fixed and stained with GFP antibodies and DAPI (blue, nuclei), and examined by fluorescence microscopy. Scale bar, 50 μm. (**C**) Determination of EGFP fluorescence in iPAM cells transfected and treated by Gen 5 software. The results represent the inhibition of EGFP expression compared with EGFP expression in the untreated corresponding control. (**D**) CCK-8-based cell viability assay for PDS as described in Materials and Methods. Data are shown as means and standard deviations of data from three independent experiments. *P < 0.05; ***P < 0.001; ****P < 0.0001.

To investigate the potential biological function of the PRRSV-G4, we constructed a series of plasmid constructs by inserting the PRRSV-G4 sequence or its mutant sequence in a vector just 5′ of the EGFP gene, immediately after the ATG start codon (Figure 3A). iPAM cells were transfected with the plasmids and then treated with PDS for 12 h. Confocal fluorescence assay indicated that cells expressing the construct harboring the PRRSV-G4 or HCV G4 sequence showed reduced fluorescence after PDS treatment compared with untreated corresponding control cells, while the reduction was attenuated in cells expressing PRRSV-G4 or HCV G4 mut under the same circumstances (Figure 3B). Quantitative evaluation by cell imaging multi-mode reader cytation 5 further confirmed these results. PDS treatment reduced EGFP expression in cells transfected with pEGFP-PRRSV-G4 or pEGFP-HCV-G4. In contrast, PDS treatment had less of an effect on EGFP expression in the cells transfected with pEGFP-PRRSV-G4 mut or pEGFP-HCV-G4 mut (Figure 3C). No cytotoxicity was observed in the cells treated with PDS at the concentrations used in our experiments (Figure 3D). Taken together, these results show that PRRSV-G4 stabilization may correspond to the suppression of EGFP reporter gene expression.

### PDS inhibits PRRSV infection *in vitro*

**Figure 4.**
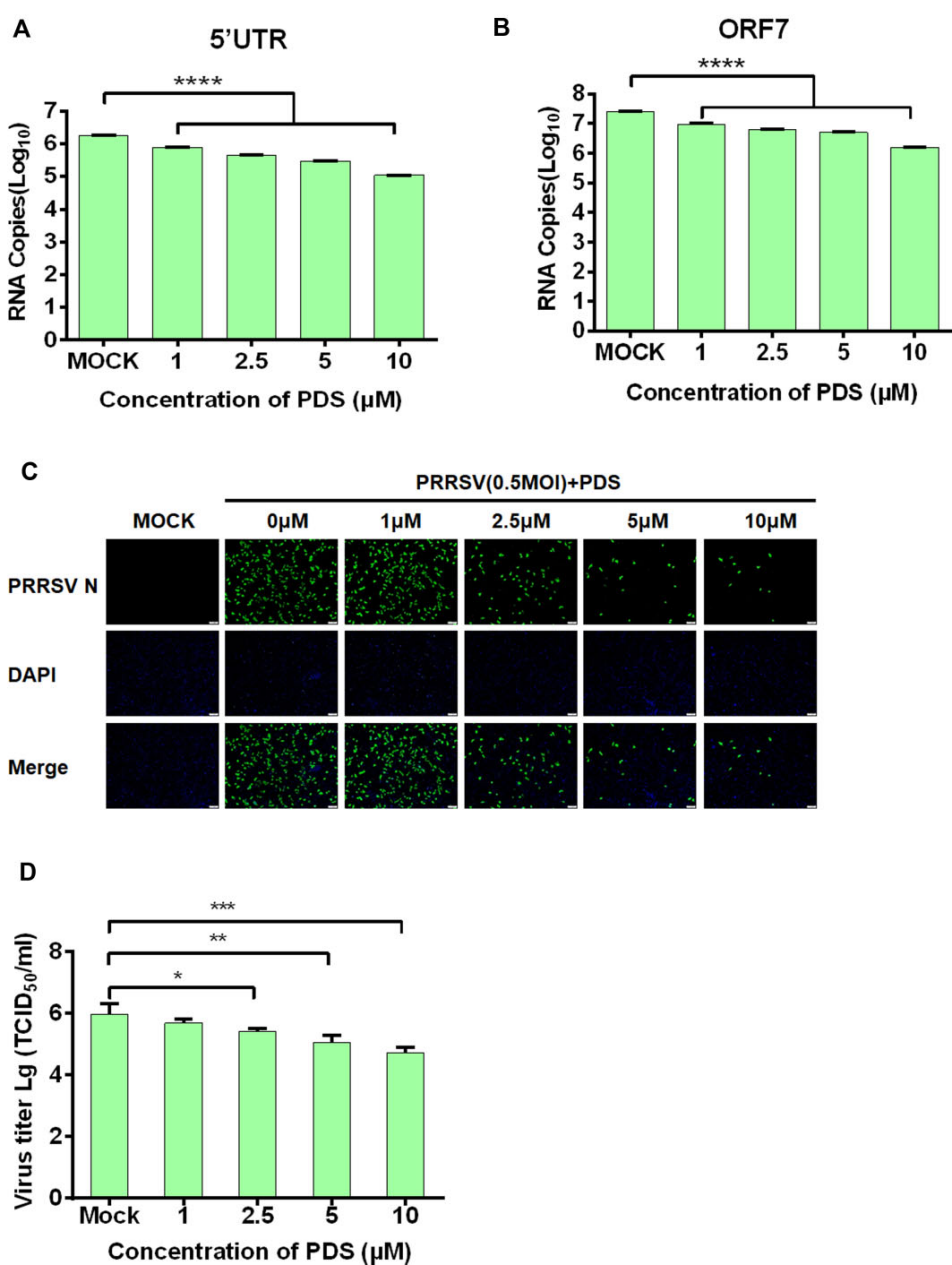
PDS suppresses PRRSV replication. iPAM cells were treated with increasing concentrations of PDS and then infected with PRRSV at a MOI of 0.5 for 12 h, followed by the evaluation of viral replication by RT-qPCR (**A, B**), IFA (**C**) and TCID_50_ assays (**D**) for detecting mRNA levels of PRRSV 5′UTR and ORF7, viral N protein expression, and virus titers, respectively. Scale bar, 100 μm. The presented results represent the means and standard deviations of data from three independent experiments. *P < 0.05; **P < 0.01; ***P < 0.001; ****P < 0.0001.

The negative strand genome RNA is required for efficient RNA replication of PRRSV. Therefore, we hypothesized that the PRRSV-G4 may play vital roles in regulating virus replication. We next examined the effect of PDS on PRRSV infection in iPAM cells by RT-qPCR, western blot, IFA, and TCID_50_ assay. At the same time, we also detected the mRNA and protein expression of three host genes (sirtuin 2, SIRT2; vacuolar protein sorting, VPS35; glucose-regulated protein 94, GRP94) with potential G4 forming sequences as additional controls. RT-qPCR results displayed a dose-dependent reduction in the subgenomic RNAs and negative strand genome RNA levels of PRRSV with increasing concentrations of PDS compared with controls (Figure 4A and B). However, PDS did not inhibit the mRNA and protein expression of SIRT2, VPS35 and GRP94 (Supplementary Figure S4). Consistent with the results of RT-qPCR, the results from IFA and TCID_50_ assay confirmed that PDS mildly inhibited PRRSV infection, evidenced by the reduction of N protein expression and virus titers, respectively, in a dose-dependent manner (Figure 4C and D). In addition, we used a drug inhibitor, Lovastatin, which targets the 3-hydroxy-3-methylglutaryl coenzyme A reductase (HMGCR) to inhibit the synthesis of cholesterol and PRRSV infection (55), to compare the antiviral effect of PDS with Lovastatin. Cell viability assay for Lovastatin showed that no obvious cytotoxicity could be observed when the concentration was up to 20 μM (Supplementary Figure S5A). As shown in Supplementary Figure S5B, Lovastatin inhibited PRRSV replication, but displayed a significantly weaker antiviral effect compared with PDS at the same concentration. We further investigated which step(s) of the viral life cycle are inhibited by PDS, including viral adsorption, internalization, replication and release steps. The results showed that PDS inhibited viral replication step, but not the adsorption, internalization and release steps. Moreover, PDS did not directly inactivate PRRSV particles *in vitro* (Supplementary Figure S6). Taken together, these findings provide evidence that PDS treatment suppresses PRRSV infection, suggesting that the stabilization of PRRSV-G4 negatively modulates viral replication.

### Host DDX18 and viral nsp10 helicases unfold the PRRSV-G4

Considering that formation of the G4 structure in the negative strand genome RNA of PRRSV negatively impacted the replication of PRRSV, we speculated that PRRSV may require RNA helicases to destabilize G4 structures to enable viral replication. To explore RNA helicases that may function to unwind the PRRSV-G4, we performed screening of proteins that interact with PRRSV-G4 in PRRSV-infected cells using streptavidin pull down assays (SPDA) and mass spectrometry. The results identified several helicases among the proteins enriched in PRRSV-G4 pulldown samples compared with controls (Supplementary data 2). Among the identified proteins, we focused on DDX18, which belongs to a subfamily of helicase superfamily 2 (SF2), as it has been reported to be associated with PRRSV replication (56). Although the PRRSV-encoded helicase (nsp10) was not hit in the SPDA/mass spectrometry data, given that nsp10 has RNA unwinding activity and is the sole helicase encoded by PRRSV (46), we also investigated the role of nsp10 on the unfolding of PRRSV-G4 in subsequent experiments.

CD spectroscopy has been used to characterize the conformational changes of G4 structures in solution and the effect of proteins on the conformation of the G4s (57,58). Previous studies showed that the presence of ATP plays an important role in perturbing the higher-order structure of nucleic acids by the helicase (59,60). Thus, we investigated the effect of purified DDX18 and nsp10 on the PRRSV-G4 or HCV G4 and PRRSV-G4 mut as controls in the absence or presence of ATP by CD spectroscopy (61,62). GST protein served as a negative control (Supplementary Figure S7A). In addition, we obtained purified mutant proteins DDX18-K345E and nsp10-E226A (Supplementary Figure S7A), and confirmed that DDX18-K345E possesses a weaker ATP hydrolase activity compared to wild-type DDX18 via ATPase hydrolysis experiments (data not shown). DDX18-K345E and nsp10-E226A were used as additional controls in this experiment. The results showed that the molar ellipticity at 260 nm decreased upon the addition of DDX18 and nsp10, and further slightly decreased with the addition of ATP (Supplementary Figures S7B and S7C). GST did not exhibit such effect on molar ellipticity at 260 nm of PRRSV-G4 in the absence or presence of ATP (Supplementary Figure S7D). Effects of DDX18 and nsp10 on HCV G4 and PRRSV-G4 mut structure are negligible in the absence or presence of ATP compared to that of PRRSV-G4 (Supplementary Figure S7E-H). In addition, DDX18-K345E and nsp10-E226A exhibited a weaker effect on PRRSV-G4 structure compared with wild-type DDX18 and nsp10, as demonstrated by the lesser decrease in molar ellipticity at 260 nm (Supplementary Figures S7I and S7J). These results suggest that DDX18 and nsp10 interact with and impact the PRRSV-G4 structure in an ATP-dependent manner.

**Figure 5.**
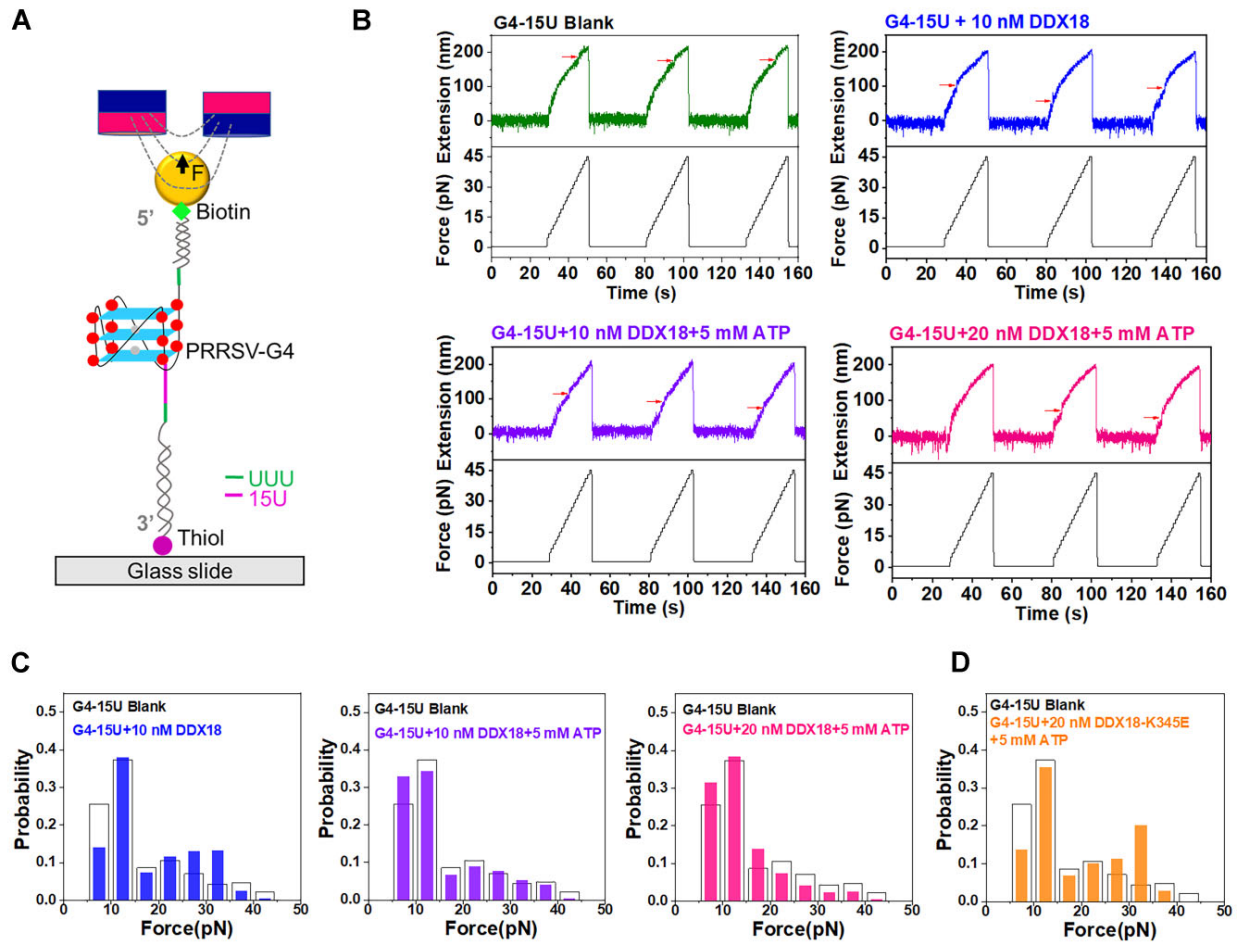
Evaluation of the unfolding of PRRSV-G4 structure by DDX18 using single-molecule magnetic tweezers. (**A**) Schematic diagram of the magnetic tweezers setup and the G4-15U construct. The G4 (dark red for guanine, light blue for G-quartet) and a poly 15U at the 3′ end (magenta) is flanked by two RNA-DNA hybrid strand handles and tethered between a paramagnetic bead and a coverslip. G4-15U has a UUU (cyan) base at each end to provide enough space for G4 structure folding. (**B**) Force extension curve of G4-15U in buffer, 10 nM DDX18, 10 nM DDX18 and 5 mM ATP, and 20 nM DDX18 and 5 mM ATP. (C, D) The unfolding force distribution of G4-15U in buffer (blank column), 10 nM DDX18 (blue column), 10 nM DDX18 and 5 mM ATP (purple column), and 20 nM DDX18 and 5 mM ATP (magenta column) (**C**), 20 nM DDX18-K345E and 5 mM ATP (orange column) (**D**).

To further confirm the effect of DDX18 and nsp10 on the unfolding kinetics of PRRSV-G4, we performed single-molecule magnetic tweezers experiments. Previous studies suggested that a 3′ ssDNA/ssRNA tail facilitates DEAH-box helicase unfolding activity (63,64). We thus introduced a 15-nt single-stranded uracil tail (U-tail) at the 3′ end of the G4 forming sequence to facilitate DDX18 activity. We denoted the RNA-DNA hybrid strand construct with the 15 nt U-tail by G4-15U (Figure 5A). Constant loading force experiments were performed with forces ranging from 5–50 pN at a constant rate of 2 pN/s (Figure 5A). Typical force extension curves for the G4-15U construct before and after introduction of different concentrations of DDX18 with or without ATP are shown in Figure 5B. Red arrows indicated a sudden change in the curve with increasing force as the G4-15U structure unfolded. Data in Figure 5C show the unfolding force distribution through calculating the unfolding force corresponding to each unfolding event after multiple cyclic stretching of more than three different beads. These results showed that the addition of DDX18 did not result in the reduction of G4-15U stability without ATP (Figure 5C). Upon the addition of ATP, the unfolding force distribution of the G4-15U shifted to lower forces, indicative of an ATP-dependent destabilization of the G4-15U by DDX18. When a higher concentration of DDX18 was introduced in the presence of ATP, we observed a continual left shift of the unfolding force distribution of the G4-15U, demonstrating that the stability of the G4-15U was further attenuated. Furthermore, we detected the effect of DDX18-K345E (20 nM) on the unfolding kinetics of PRRSV-G4 in the same condition as described in Figure 5C. The results showed that DDX18-K345E resulted in a reduced left shift of the unfolding force distribution of the G4-15U compared with wild-type DDX18 (Figure 5D), further demonstrating that DDX18 unfolded the PRRSV-G4 in an ATP-dependent manner. Different from DDX18, a 15-nt single-stranded uracil tail (U-tail) was introduced at the 5′ end of the G4-forming sequence to facilitate nsp10 activity because of the translocation polarity of SF1B family helicases (46); the resulting RNA-DNA hybrid strand construct with the 15 nt U-tail was named 15U-G4. Similar results were obtained in experiments using purified nsp10 and its mutant nsp10-E226A, suggesting that nsp10 unfolds the 15U-G4 in a manner dependent on ATP (Supplementary Figure S8). Taken together, these results indicate that host DDX18 and viral nsp10 possess the ability to unfold the PRRSV-G4 structure in an ATP-dependent manner.

### Host DDX18 and viral nsp10 promote PRRSV replication

**Figure 6.**
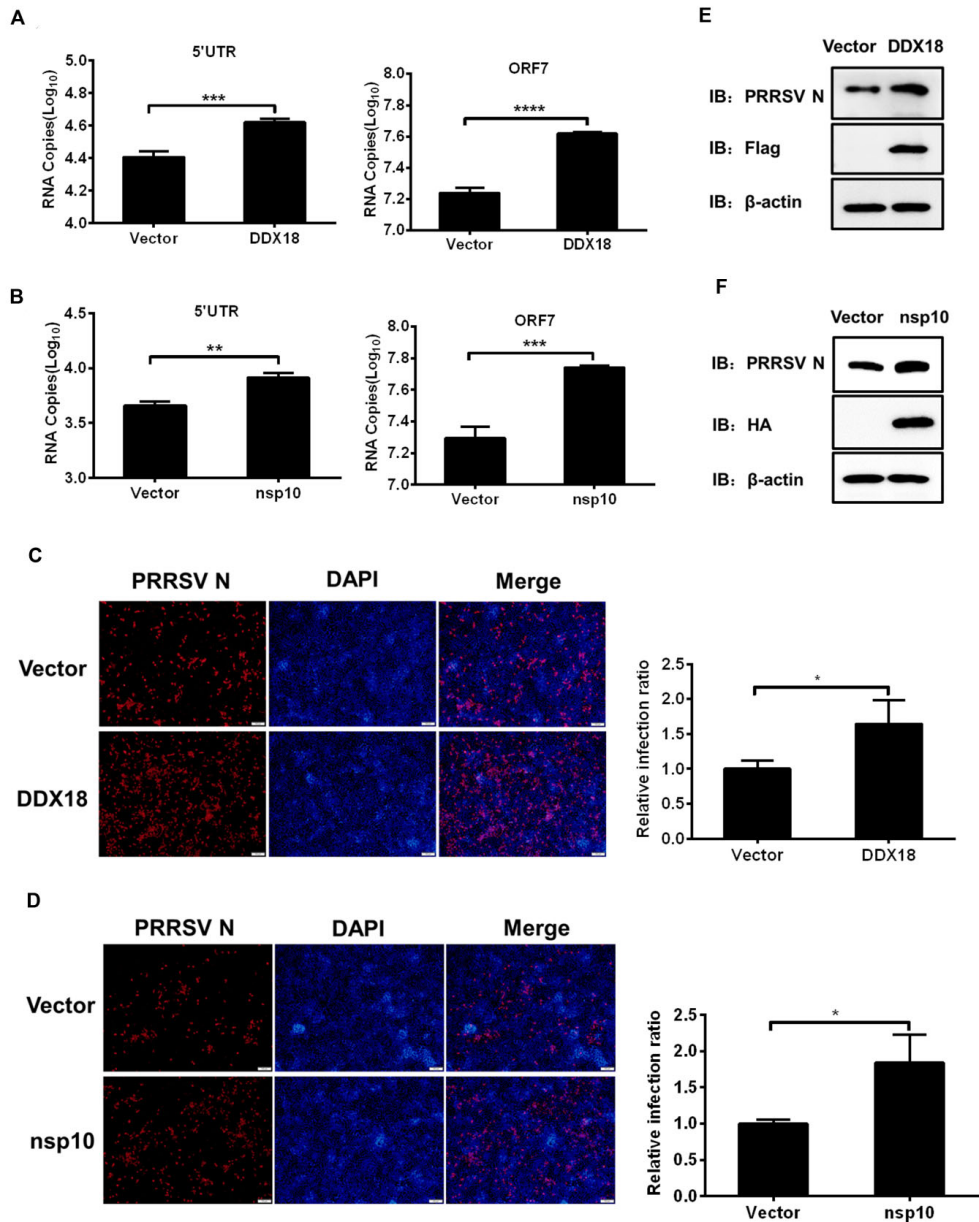
DDX18 and nsp10 promote PRRSV replication *in vitro*. PK-15^CD163^ (**A–D**) and iPAM (**E**, **F**) cells were transfected with expression constructs encoding DDX18 or nsp10, respectively, and then infected with PRRSV (MOI = 0.1) for 24 h, followed by the evaluation of viral replication via RT-qPCR (A, B), IFA (C, D) and western blot (E and F) for detecting mRNA levels of PRRSV 5′UTR and ORF7 and viral N protein expression levels. Scale bar, 100 μm. Relative viral infection ratio in panel C and D was calculated by setting the values of vector control group to 1.0 for statistical analyses. The presented results represent the means and standard deviations of data from three independent experiments. *P < 0.05; **P < 0.01; ***P < 0.001; ****P < 0.0001.

Our results showed that DDX18 and nsp10 both unwind the PRRSV-G4 structure. We thus next evaluated the effect of the two helicases on PRRSV replication by RT-qPCR, IFA, and western blot. The results indicated that overexpression of DDX18 and nsp10 promoted viral genomic replication as evidenced by the increase in the RNA levels of PRRSV 5′UTR and ORF7 (Figure 6A and B). In line with the RT-qPCR results, IFA and western blot further confirmed that DDX18 and nsp10 increased viral protein expression (Figure 6C–F). CCK-8 and EdU assays results showed that overexpression of the DDX18 and nsp10 had no obvious effects on cell viability and cell proliferation in PK-15 CD^163^ and iPAM cells compared to control group (Supplementary Figure S9). These results showed that host DDX18 and viral nsp10 unwind the PRRSV-G4 structure, thereby promoting viral replication.

### Recombinant PRRSV with G4-disruptive mutation displays resistance to the antiviral activity of PDS

**Figure 7.**
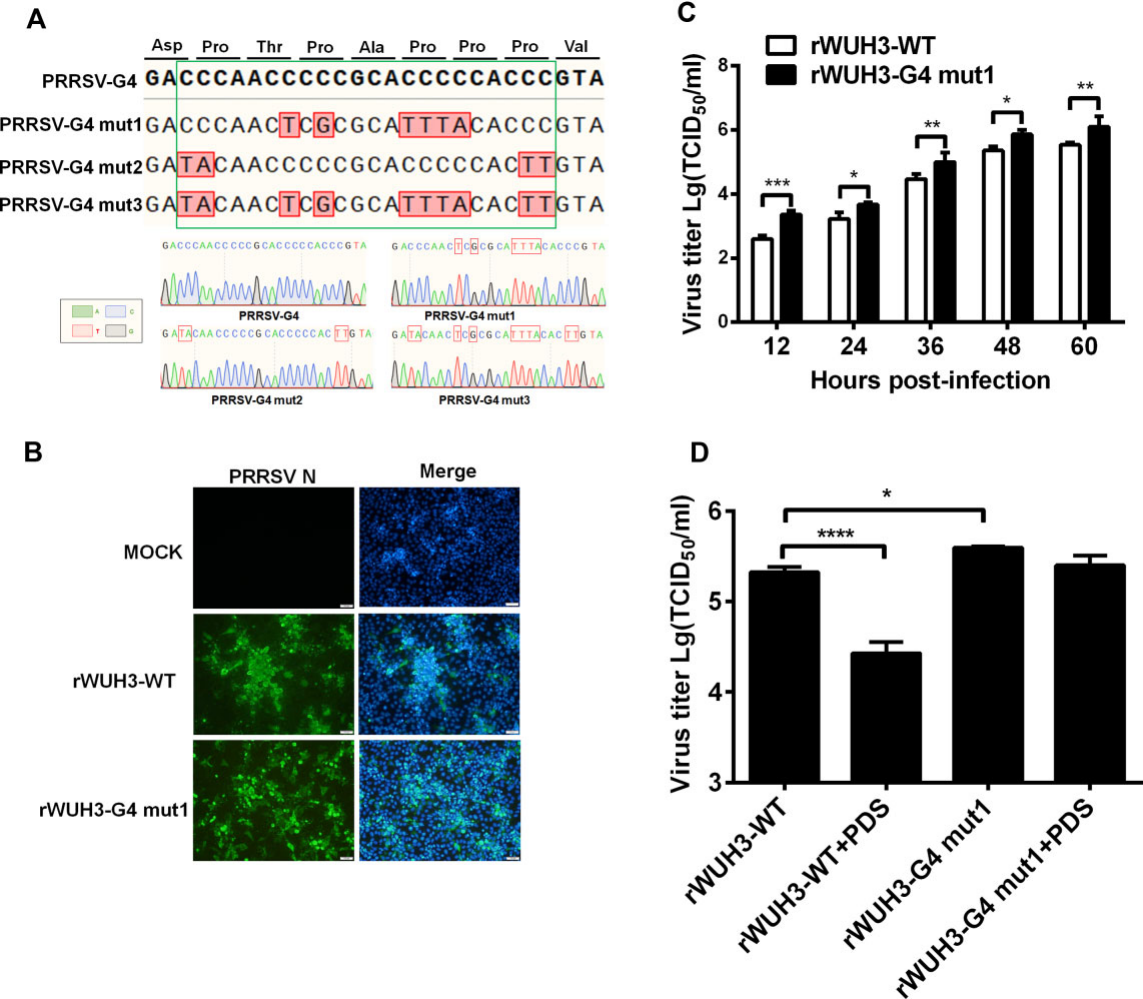
Recombinant PRRSV with G4-disruptive mutation displays resistance to the antiviral activity of PDS. (**A**) Schematic diagram of G4-disruptive mutations with an amino acid extension at each end, and identification of recombinant plasmids with G4-disruptive mutation by Sanger sequencing. Green frame indicates PRRSV-G4 and the mutants. Nucleotides highlighted in red (filled box and frame) are mutated bases. (**B**) IFA of MARC-145 cells infected with rWUH3-WT and rWUH3-G4 mut1. (**C**) Multiple-step growth curves of rWUH3s in MARC-145 cells. Cells were infected with rWUH3s (MOI = 0.1) and then collected at the different time points post-infection (12, 24, 36, 48, 60 hpi) and subjected to a TCID_50_ assay. (**D**) iPAM cells were pre-treated with PDS and then infected with rWUH3s (MOI = 0.5) for 12 h, followed by a TCID_50_ assay. The presented results represent the means and standard deviations of data from three independent experiments. *P < 0.05; **P < 0.01; ***P < 0.001; ****P < 0.0001.

To further confirm the contribution of the PRRSV-G4 to PDS-mediated antiviral activity, we next constructed recombinant PRRSV with the G4-disruptive mutation. By referencing the multiple sequence alignment results, we mutated partial G to other bases that occurred in other PRRSV strains in the corresponding sites for destroying the G4 structure, ensuring the greatest potential to reduce lethal mutations. CD spectroscopy and native PAGE analysis showed that two G4 mutants (PRRSV-G4 mut1 and PRRSV-G4 mut3) led to complete destruction of the G4 structure, similar to the positive control PRRSV-G4 mut (Supplementary Figure S10). Compared with the PRRSV-G4 mut, the PRRSV-G4 mut2 showed significantly attenuated G4 structure formation ability (Supplementary Figure S10). We constructed three recombinant plasmids with the indicated G4-disruptive mutations (Figure 7A). From these constructs, we only successfully generated recombinant PRRSV containing the PRRSV-G4 mut1 sequence, designated as rWUH3-G4 mut1, demonstrated by IFA results (Figure 7B). TCID_50_ assays demonstrated that the titres of rWUH3-G4 mut1 were slightly higher than those of wild-type rPRRSV (rWUH3-WT) (Figure 7C). These results suggest that the PRRSV-G4-disruptive mutation enables increased viral replication. To verify this result, iPAM cells were treated with PDS and then infected with rWUH3-WT and rWUH3-G4 mut1, followed by TCID_50_ assay. As shown in Figure 7D, PDS significantly suppressed the replication of rWUH3-WT; however, the inhibitory effects of PDS were attenuated in rWUH3-G4 mut1-infected cells. Taken together, these results indicate that recombinant PRRSV with the G4-disruptive mutation displays resistance to the antiviral activity of PDS.

## DISCUSSION

Despite the considerable progress in understanding the infection, immunity, pathogenesis of PRRSV over the past decades (55,65–68), effective methods for control of this virus have not been established. New areas for PRRS control and prevention remain to be further explored. In this study, we confirmed the presence of a highly conserved G-rich sequence with parallel-type G4 (PRRSV-G4) in the negative strand genome RNA of PRRSV. PDS targeted the G4 structure and inhibited PRRSV replication. We further identified two helicases, host DDX18 and viral nsp10, that interact with PRRSV-G4 and unwind the G4 structure to facilitate viral replication. Recombinant PRRSV with a G4-disruptive mutation displayed resistance to the antiviral activity of PDS. Together our findings suggest that PRRSV-G4 may serve as a potential target for antiviral therapy.

A previous study showed that an RNA G4 sequence within the open reading frame of a gene inhibits the translation of mRNA into protein (69). In the present study, wild-type or G4-mutant sequences were fused into the N terminus of an EGFP reporter gene and used to investigate whether PDS functions as a specific inhibitor. PDS treatment significantly inhibited the expression of EGFP in the constructs with wild-type G4 sequence, while EGFP expression in the constructs with G4-disruptive mutations was relatively insensitive to PDS treatment. We further evaluated whether PDS has antiviral activity against PRRSV and confirmed that PDS significantly suppressed PRRSV replication at both RNA and protein levels. To further validate G4 as a target of PDS, we successfully generated a recombinant PRRSV with a G4-disruptive mutation. Compared with rWUH3-WT, rWUH3-G4 mut1 exhibited slightly higher replication and resistance to PDS treatment (Figure 7C and D), confirming that PDS targeted the G4 structure and inhibited PRRSV replication in the context of viral infection. We speculate that the failure to obtain the other two recombinant virus with G4-disruptive mutations may have been from the amino acid alteration generated by the G4-disruptive mutation (Figure 7A). In agreement with our results, several studies showed that PDS targets and stabilizes G4 structures to reduce replication of viruses such as HCV, Epstein–Barr virus, and SARS-CoV-2 (10,70).

The replication of genome frequently encounters many barriers that must be overcome (71). For example, nucleic acid sequences form secondary structures that act as barriers to replication and potential inducers of genome instability (72). Helicases are thus required for overcoming some of these structural barriers through their functions in hairpin unwinding and fork progression (71). Most studies on helicase family proteins and their functions with respect to G4 structures have mainly focused on DNA G4 structures, while few have addressed their activities towards RNA G4 structures (73–77). In this study, we confirmed that purified wild-type DDX18 and nsp10 proteins both unwind the PRRSV-G4 structure through CD spectroscopy and single-molecule magnetic tweezers analysis (Supplementary Figures S7 and S8; Figure 5). In contrast, ATPase activity-inactive DDX18 and nsp10 mutants exhibit a weaker effect on the unfolding of PRRSV-G4 structure compared with wild-type DDX18 and nsp10 (Supplementary Figures S7 and S8; Figure 5), supporting this conclusion that DDX18 and nsp10 could unwind the PRRSV-G4 structure in an ATP-dependent manner. DEAD-box/DEAH-box helicases (DEAD/H-box helicases), which belong to a subfamily of helicase superfamily 2 (SF2), recognize and unwind RNA duplexes and displace bound protein complexes in an ATP-dependent manner (78). Many DEAD/H-box helicases, such as DHX36, DHX9, DDX21, DDX1 and DDX3X, have been reported to unwind G4s in DNA or RNA sequences (75–77,79). In the SPDA/mass spectrometry data, we detected some DDXs, such as DDX18, DDX3X, DDX1, DDX23, DDX24, DDX54 and so on. Among them, DDX18 had the most 24 peptides to be detected in the SPDA/mass spectrometry data with the highest coverage approximately 38% of full-length DDX18 (Supplementary data 2). Importantly, DDX18 has been confirmed to positively regulate PRRSV replication via interacting with nsp2 and nsp10 (56), but the detailed mechanism is unclear. Thus, we selected DDX18 as an object of study and expected to elucidate its action mechanism to promote PRRSV replication. Our results may provide an explanation for the mechanism by which DDX18 promotes PRRSV replication. In addition, previous studied showed that PRRSV-encoded helicase (nsp10) has both RNA and DNA duplex unwinding activity, similar to SARS-CoV-encoded helicase (nsp13) (46,80). A recent study identified G4s in SARS-CoV-2 and confirmed their recognition and unwinding by a viral helicase (nsp13) (81). Although PRRSV nsp10 was not hit in the SPDA/mass spectrometry data, the results of CD spectroscopy and single-molecule magnetic tweezer experiments demonstrated that nsp10 could bind and unfold the PRRSV-G4 (Supplementary Figures S7 and S8). We speculate that viral nsp10 is not efficient enough to unwind PRRSV-G4, and thus PRRSV hijacks the host protein DDX18 to unfold G4 for viral replication. However, we cannot not rule out the possibility that other host helicases and virus-encoded proteins are also involved in the unwinding of the PRRSV-G4 structure.

Together, our results suggest that PRRSV employs its own helicase nsp10 and hijacks the host DDX18 helicase to unwind the PRRSV-G4 structure to enhance transcription and replication of the viral genome. This is the first report that both a host and viral helicase are implicated in the unwinding of the RNA G4 structure in a virus genome. The identification and characterization of helicases and G4 structures provide a new direction for analyzing the viral pathogenic mechanism and exploring treatment strategies. The specific functional mechanism of helicases in virus infection remains to be further investigated.

In conclusion, this is the first report describing the presence of a conserved G-rich sequence that forms a G4 structure within the negative strand genome of PRRSV. We demonstrated that the G4 ligand PDS specifically targets the G4 structure to inhibit viral replication. PRRSV uses both host helicase DDX18 and viral nsp10 to unfold the G4 structure and facilitate viral replication. These findings suggest that targeting the viral RNA G4 structure and helicases represents a promising new strategy for the design and development of anti-PRRSV compounds.

## Supplementary Material

gkad759_Supplemental_Files

## Data Availability

The data underlying this article are available in this manuscript and in its online supplementary material.
