# Supplementary material for: Unfolding of an RNA G-quadruplex motif in the negative strand genome of porcine reproductive and respiratory syndrome virus by host and viral helicases to promote viral replication
Source: Nucleic Acids Res. 2023 Sep 22;51(19):10752–67. doi: 10.1093/nar/gkad759 (PMC10602871; doi:10.1093/nar/gkad759)
Supplement: gkad759_Supplemental_Files [file gkad759_supplemental_files.zip › Supplementary data 1.pdf]

SUPPLEMENTARY DATA for

Unfolding of an RNA G-quadruplex motif in the negative strand genome of porcine reproductive and respiratory syndrome virus by host and viral helicases to promote viral replication

Puxian Fang[1,2,†], Congbao Xie[1,3,4,5†], Ting Pan[1,2,†], Ting Cheng[1,2], Wei Chen[1,2], Sijin Xia[1,2], Tong Ding[1,2], Junkang Fang[1,3,4,5], Yanrong Zhou[1,2], Liurong Fang[1,2], Dengguo Wei[1,3,4,5,*], Shaobo Xiao[1,2,*]

[1]National Key Laboratory of Agricultural Microbiology, College of Veterinary Medicine, Huazhong Agricultural University, Wuhan 430070, China
[2]The Key Laboratory of Preventive Veterinary Medicine in Hubei Province, Cooperative Innovation Center for Sustainable Pig Production, Wuhan 430070, China
[3]Hubei Hongshan Laboratory, and Interdisciplinary Sciences Institute, Huazhong Agricultural University, Wuhan 430070, China
[4]Shenzhen Institute of Nutrition and Health， Huazhong Agricultural University, Shenzhen 518000, China,
[5]Shenzhen Branch, Guangdong Laboratory for Lingnan Modern Agriculture, Genome Analysis Laboratory of the Ministry of Agriculture, Agricultural Genomics Institute at Shenzhen, Chinese Academy of Agricultural Sciences, Shenzhen 518000, China

[†]These authors contribute equally to this work and should be considered co-first authors.
[*]To whom correspondence should be addressed. Tel: +86 27 87286884; E-mail: vet@mail.hzau.edu.cn (S.X.); dgwei@mail.hzau.edu.cn (D.W.)

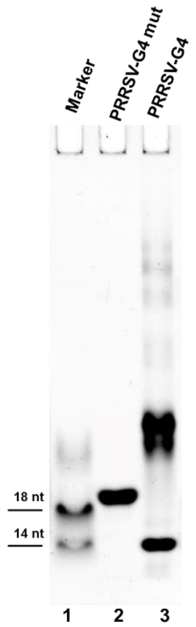

Figure S1. Native PAGE assay for characterizing the G4 structure of PRRSV-G4 wild-type and mutant. A total 25 pmol of annealed RNA was loaded in each lane. Lane 1, ssRNA markers; Lane 2, PRRSV-G4 mut; Lane 3, PRRSV-G4.

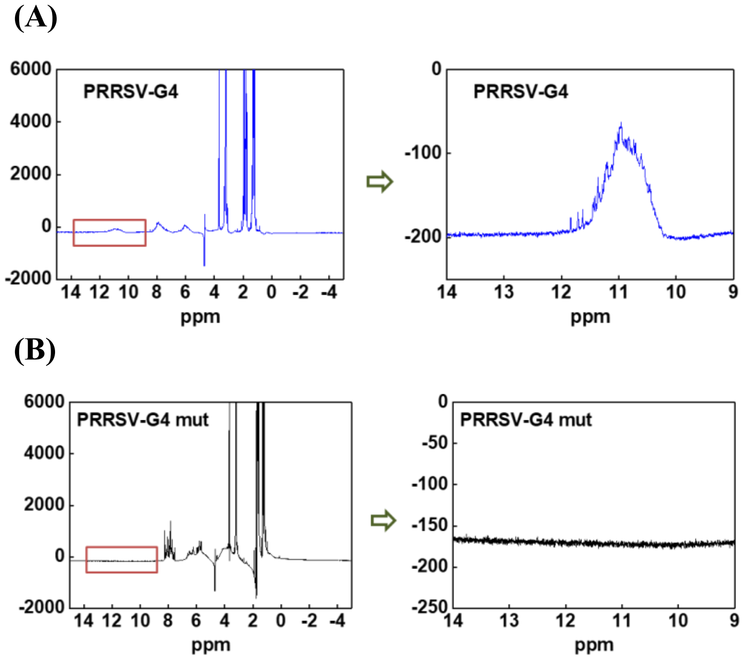

Figure S2. Synthetic PRRSV-G4 sequence forms a G4 structure. (**A**) ^{1}H NMR spectra of PRRSV-G4 (left) and its imino region signal (right). (**B**) ^{1}H NMR spectra of PRRSV-G4 mut (left) and its imino region signal (right). RNA samples were dissolved in 5 mM Kcaco (pH=6.5), 50 mM KCl, and 10% D2O at a final concentration of 300 μM.

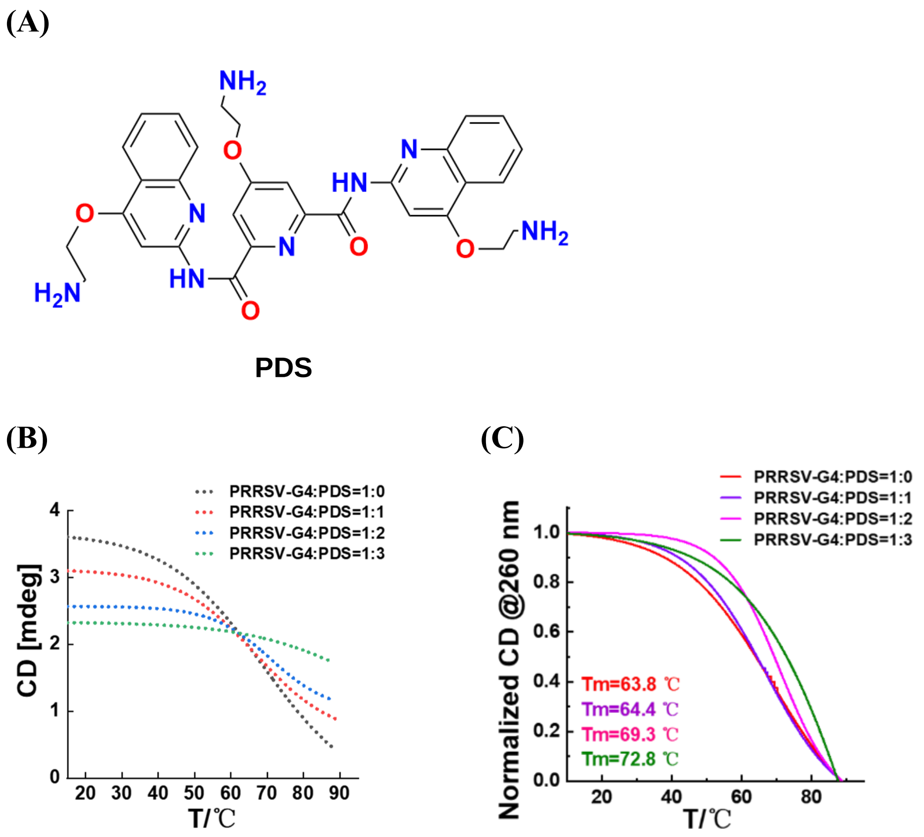

Figure S3. PDS stabilizes the PRRSV-G4 RNA structure. (**A**) The chemical structure of the compound PDS. (**B**) The melting curve of PRRSV-G4 in the presence of different concentrations of PDS, λ=260 nm, T=15-90 °C (T_{step}=1°C). (**C**) The normalized data in panel B. Experiments were performed at the concentration of 5 μM RNA with different concentrations of PDS in 10 mM Tris-HCl buffer (pH 7.4) supplemented with 5 mM KCl.

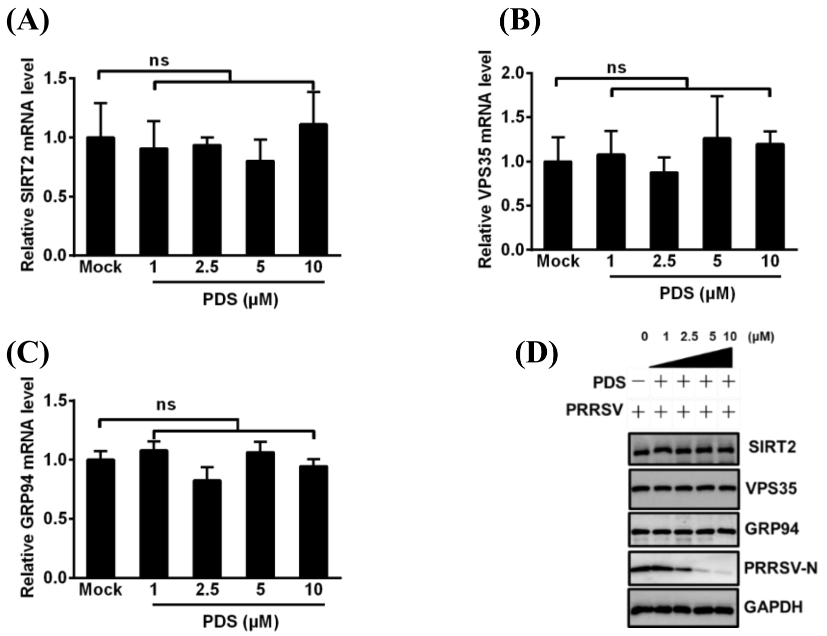

Figure S4. Effects of PDS on the expression of three host genes (SIRT2, VPS35 and GRP94) with potential G4 forming sequences. iPAM cells were treated with increasing concentrations of PDS and then infected with PRRSV at a MOI of 0.5 for 12 h, followed by the evaluation of the mRNA and protein expression of SIRT2, VPS35 and GRP94 by RT-qPCR (**A-C**) and western blot assay (**D**), respectively. The presented results represent the means and standard deviations of data from three independent experiments. ns, nonsignificant difference.

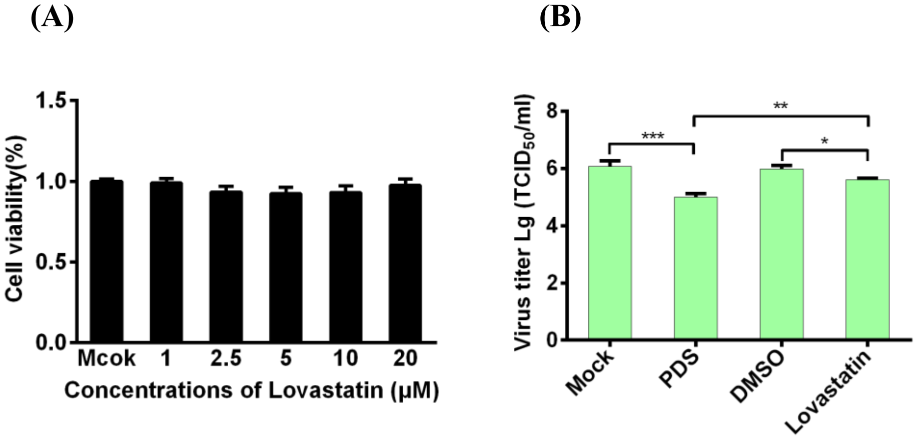

Figure S5. Comparative effect of PDS and Lovastatin on PRRSV infection. (**A**) CCK-8-based cell viability assay for Lovastatin as described in Materials and Methods. (**B**) iPAM cells were treated with the same concentrations (10 μM) of PDS and Lovastatin then infected with PRRSV at a MOI of 0.5 for 12 h, followed by the evaluation of viral replication by $TCID_{50}$ assays. The presented results represent the means and standard deviations of data from three independent experiments. *p < 0.05; **p < 0.01; ***p < 0.001.

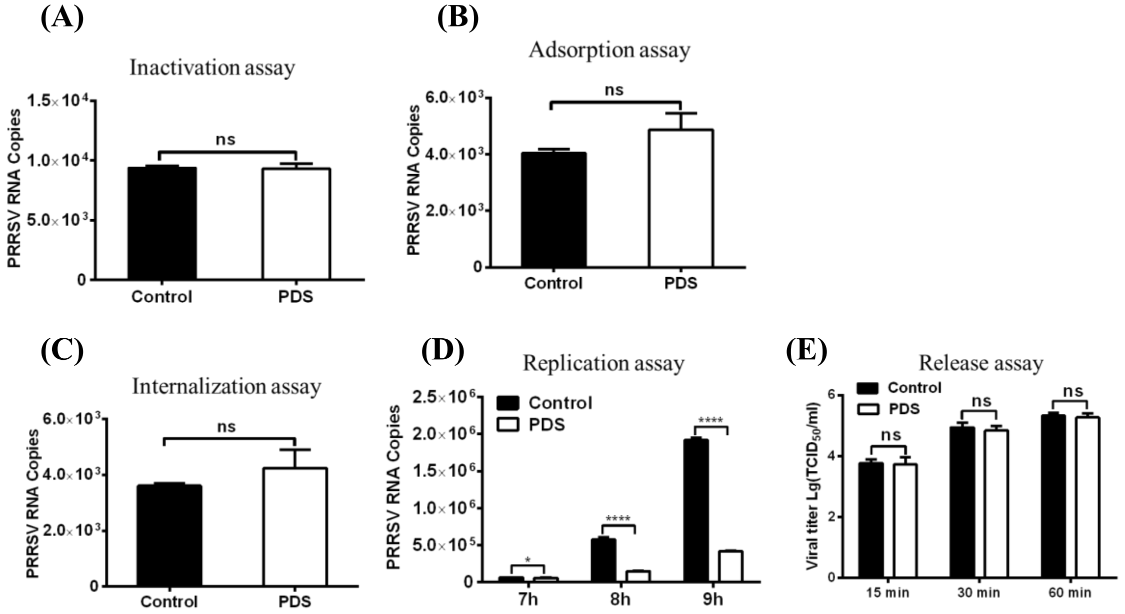

Figure S6. PDS inhibits the replication step of PRRSV infection. (**A**) PRRSV and PDS (10 μM) were incubated for 2 h and then infected iPAM cells. An absolute RT-qPCR assay were performed to detect the infectivity of the treated PRRSV particles. (**B**) iPAM cells were infected with PRRSV (MOI = 0.5) in the absence or presence of PDS (10 μM) at 4 °C for 2 h. The cells were washed with ice-cold PBS and subjected to RT-qPCR assay. (**C**) iPAM cells were infected with PRRSV (MOI = 0.5) at 4 °C for 2 h. After twice washes with ice-cold PBS, cells were cultured at 37 °C in the absence or presence of PDS (10 μM) for 2 h, followed by RT-qPCR assay. (**D**) iPAM cells were infected with PRRSV (MOI = 0.5) at 37 °C for 2 h. The cell-free virus particles were removed and cells were cultured in fresh medium containing PDS (10 μM). At different time points post-infection (7, 8 and 9 hpi), infected cells were harvested and subjected to RT-qPCR assay. (**E**) iPAM cells were infected with PRRSV (MOI = 0.5) at 37 °C for 18 h and then treated with PDS (10 μM) for 15, 30, 60 min, respectively. Cellular supernatants were collected for a $TCID_{50}$ assay. The presented results represent the means and standard deviations of data from three independent experiments. *p < 0.05; ****p < 0.0001; ns, nonsignificant difference.

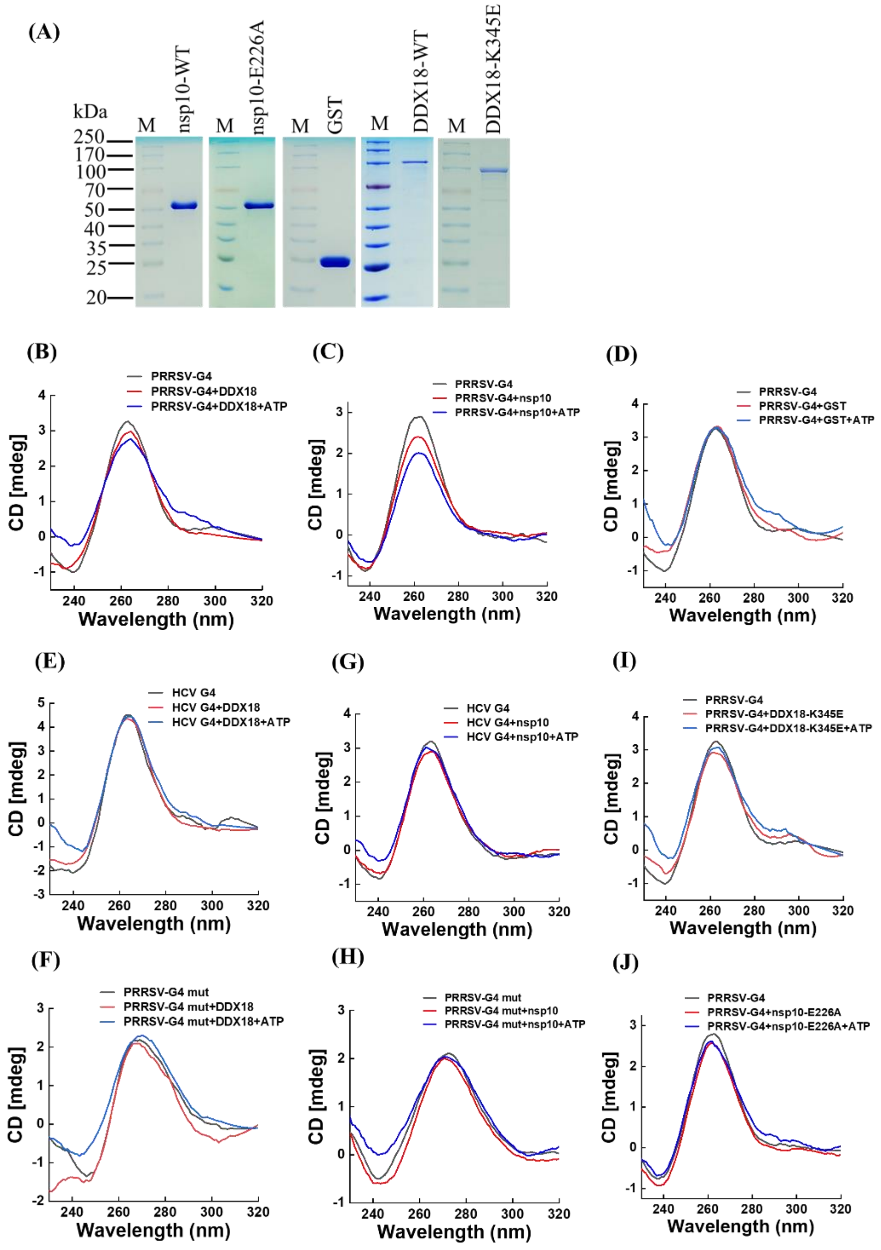

Figure S7. Effect of host DDX18, viral nsp10 and their mutants on PRRSV-G4 structure stability. (**A**) Images from SDS-PAGE analysis of purified recombinant nsp10, nsp10-E226A, GST, DDX18, and DDX18-K345E, are shown. M: protein marker. (**B–D**) The effects of DDX18 (B), nsp10 (C) and GST (D) on PRRSV-G4 structure in the absence or presence of ATP by CD spectra analysis. GST served as a negative control. (**E-H**) The effects of DDX18 (E, F), nsp10 (G, H) on HCV G4 and PRRSV-G4 structure in the absence or presence of ATP by CD spectra analysis. (**I, J**) The effects of DDX18-K345E (I), nsp10-E226A (J) on PRRSV-G4 structure in the absence or presence of ATP by CD spectra analysis. The corresponding protein buffer, protein and ATP signals have been subtracted as background. Experiments were performed at the concentration of 5 μM RNA and 5 μM helicase in 10 mM Tris-HCl buffer (pH 7.4) supplemented with 5 mM KCl and 4 mM $MgCl_2$ in the absence or presence of ATP (100 μM).

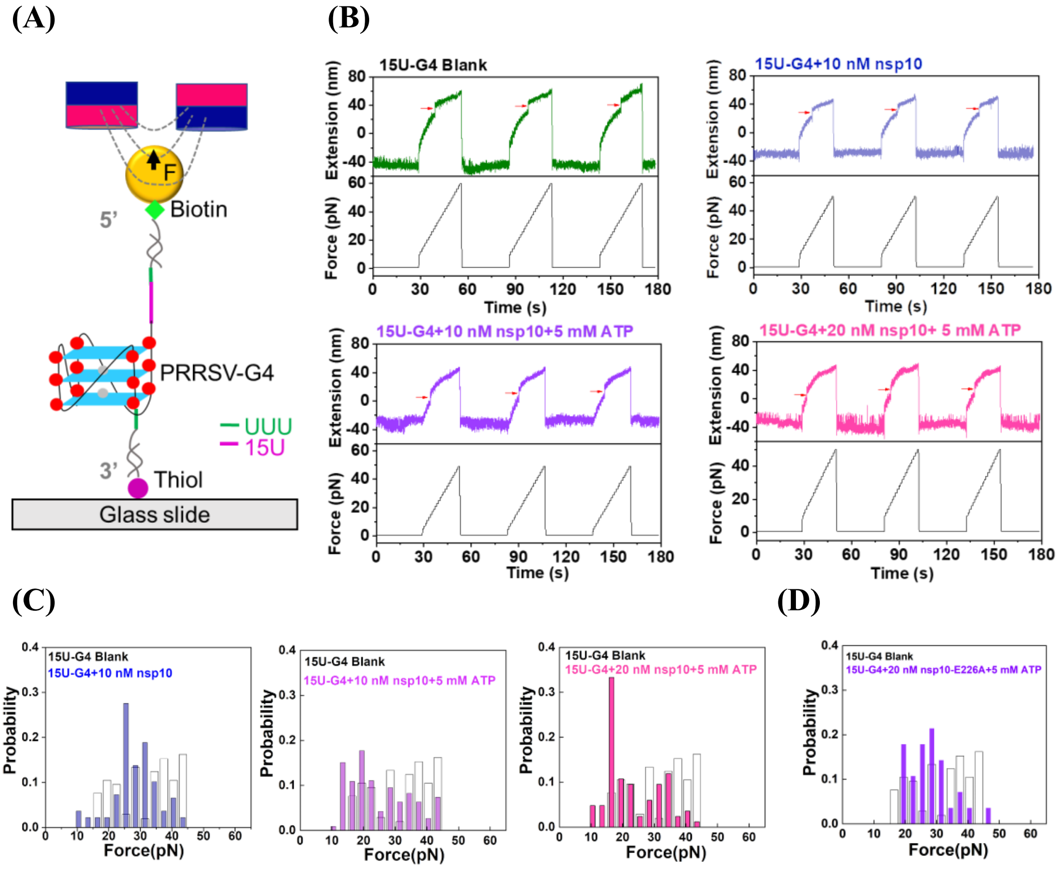

Figure S8. Unfolding of PRRSV-G4 structure by nsp10 through single-molecule magnetic tweezers. (**A**) Schematic diagram of the magnetic tweezers setup and the 15U-G4 construct. The G4 (dark red for guanine, light blue for G-quartet) and a poly 15U at the 5′ end (magenta) is flanked by two RNA-DNA hybrid strand handles and tethered between a paramagnetic bead and a coverslip. 15U-G4 has a UUU (cyan) base at each end to provide enough space for G4 structure folding. (**B**) Force extension curve of 15U-G4 in buffer, 10 nM nsp10, 10 nM nsp10 and 5 mM ATP, and 20 nM nsp10 and 5 mM ATP. (**C, D**) The unfolding force distribution of 15U-G4 in buffer (blank column), 10 nM nsp10 (blue column), 10 nM nsp10 and 5 mM ATP (light purple column), 20 nM nsp10 and 5 mM ATP (magenta column) (C), and 20 nM nsp10-E226A and 5 mM ATP (purple column) (D).

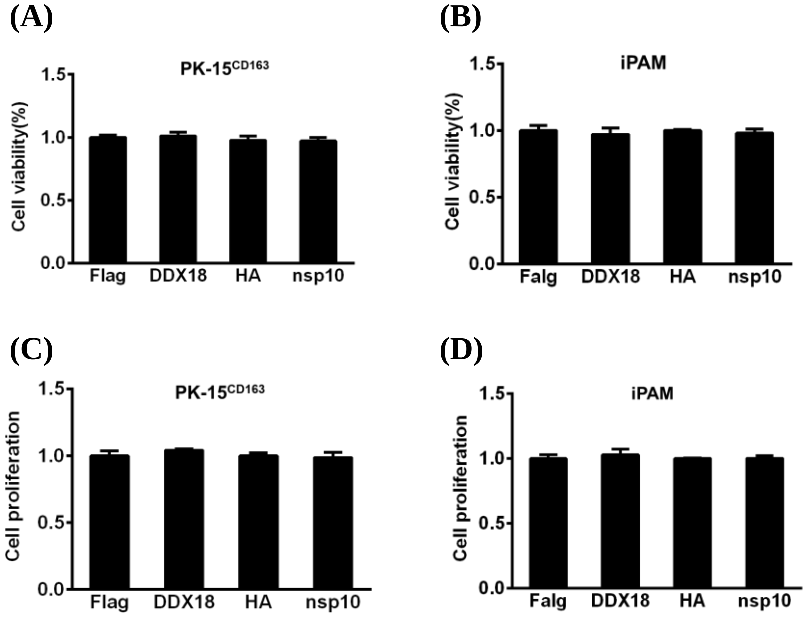

Figure S9. CCK-8 and EdU assays for detecting the effect of DDX18 and nsp10 expression on cells proliferation. PK-15CD[163] (**A, C**) and iPAM (**B, D**) cells seeded in 96-well cell culture plates were transfected with expression constructs encoding DDX18 and nsp10, or corresponding empty vector for 48 h and then cells were harvested for CCK-8 and EdU assays according to the manufacturer's protocol.

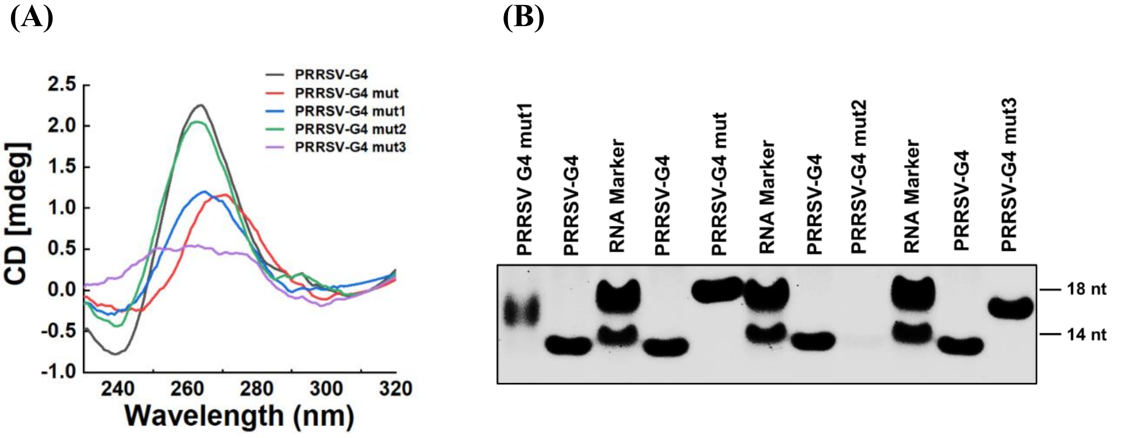

Figure S10. Determination of G4-disruptive mutations. (**A, B**) CD spectra (A) and native PAGE (B) analysis for detecting the G4 structure of PRRSV-G4 and its mutants (PRRSV-G4 mut1, PRRSV-G4 mut2, PRRSV-G4 mut3) in the presence of 100 mM K^+ ion. PRRSV-G4 mut served as a positive control.

Table S1. Primers used for plasmids construction and RT-qPCR.

Primer	Sequences (5′ to 3′)
C1-PRRSV-G4-F	TTTGCTAGCATGAGGGTGGGGGTGCGGGGGTTGGGTAGCAAG GGCGAGGAGCTGTTC
C1-PRRSV-G4 mut-F	TTTGCTAGCATGAGAGTGAAAGTGCGAAAGTTGAGTAGCAAG GGCGAGGAGCTGTTC
C1-HCV G4-F	TTTGCTAGCATGAGGGTTGCGGGTGGGCGGGATAGCAAGGGCG AGGAGCTGTTC
C1-HCV G4 mut-F	TTTGCTAGCATGAGAGTTGCGAATGAGCAAGATAGCAAGGGCG AGGAGCTGTTC
C1-R	TTTAAGCTTTTACTTGTACAGCTCGTCCATGC
nsp10-F	TTTGAATTCGGGAAGAAGTCCAGAATGTG
nsp10-R	TTTAGATCTTTCCAGGTCTGCGCAAATAG
30a-nsp10-F	ATGGCTGATATCGGATCCGGGAAGAAGTCCAGAA
30a-nsp10-R	GTGGTGCTCGAGTTATTCCAGGTCTGCGCAAAT
DDX18-F	GGCGAATTCATGTCTCATTTACCGATGA
DDX18-R	TTTCTCGAGGTGAGAGAACTGCCTGC
5’UTR-F	GCATTTGTATTGTCAGGAGC
5’UTR-R	AGCAGTGCAACTCCGGAAG
ORF7-F	GCAATTGTGTCTGTCGTC
ORF7-R	CTTATCCTCCCTGAATCTGAC
qSIRT2-F	CCCTTCGCATCCCTCAT
qSIRT2-R	ATCCCGACTGGGCATCT
qVPS35-F	GTGCGTCTCAGTCAGTTGGA
qVPS35-R	TGGTGTAACTCAGCACAGGC
qGRP94-F	TACCAGACGGGCAAGGACAT
qGRP94-R	AAGAGATACCCTGACCGCAG
qGAPDH-F	ACATGGCCTCCAAGGAGTAAGA
qGAPDH-R	GATCGAGTTGGGGCTGTGACT
sgPRRSV-1	TTAATACGACTCACTATAGGGCGCCACAGTCATTTTGGACCGTT TTAGAGCTAGA
sgPRRSV-2	TTAATACGACTCACTATAGGGAGAACGGTCCCAATGCCTGTTTT AGAGCTAGA
PRRSV-G4 mut1-F	GAGTCCTTGCCGGGTCCAAAATGACTGTGGCGCGTGTCGTTGA CCCAACTCGCGCATTTACACCCGTACCTGTGCCCATCCCTC
PRRSV-G4 mut2-F	GAGTCCTTGCCGGGTCCAAAATGACTGTGGCGCGTGTCGTTGA TACAACCCCCGCACCCCCACTTGTACCTGTGCCCATCCCTC
PRRSV-G4 mut3-F	GAGTCCTTGCCGGGTCCAAAATGACTGTGGCGCGTGTCGTTGA TACAACTCGCGCATTTACACTTGTACCTGTGCCCATCCCTC
PRRSV-G4-R	GGTCCTCATCCCCCCAGGCATTGGGACCGTTCTCCAGAACTTTC GGTGGGAGAGGGATGGGCACAGGTAC
